# Dynamic lipidome alterations associated with human health, disease and ageing

**DOI:** 10.1038/s42255-023-00880-1

**Published:** 2023-09-11

**Authors:** Daniel Hornburg, Si Wu, Mahdi Moqri, Xin Zhou, Kevin Contrepois, Nasim Bararpour, Gavin M. Traber, Baolong Su, Ahmed A. Metwally, Monica Avina, Wenyu Zhou, Jessalyn M. Ubellacker, Tejaswini Mishra, Sophia Miryam Schüssler-Fiorenza Rose, Paula B. Kavathas, Kevin J. Williams, Michael P. Snyder

**Affiliations:** 1 Department of Genetics, Stanford University, Stanford, CA USA; 2 Department of Biological Chemistry, David Geffen School of Medicine, University of California, Los Angeles, Los Angeles, CA USA; 3 Department of Molecular Metabolism, Harvard T.H. Chan School of Public Health, Boston, MA USA; 4 Departments of Laboratory Medicine and Immunobiology, Yale School of Medicine, New Haven, CT USA; 5 Lipidomics Laboratory, University of California, Los Angeles, Los Angeles, CA USA

**Keywords:** Lipidomics, Metabolomics, Mass spectrometry, Metabolic diseases, Ageing

## Abstract

Lipids can be of endogenous or exogenous origin and affect diverse biological functions, including cell membrane maintenance, energy management and cellular signalling. Here, we report >800 lipid species, many of which are associated with health-to-disease transitions in diabetes, ageing and inflammation, as well as cytokine–lipidome networks. We performed comprehensive longitudinal lipidomic profiling and analysed >1,500 plasma samples from 112 participants followed for up to 9 years (average 3.2 years) to define the distinct physiological roles of complex lipid subclasses, including large and small triacylglycerols, ester- and ether-linked phosphatidylethanolamines, lysophosphatidylcholines, lysophosphatidylethanolamines, cholesterol esters and ceramides. Our findings reveal dynamic changes in the plasma lipidome during respiratory viral infection, insulin resistance and ageing, suggesting that lipids may have roles in immune homoeostasis and inflammation regulation. Individuals with insulin resistance exhibit disturbed immune homoeostasis, altered associations between lipids and clinical markers, and accelerated changes in specific lipid subclasses during ageing. Our dataset based on longitudinal deep lipidome profiling offers insights into personalized ageing, metabolic health and inflammation, potentially guiding future monitoring and intervention strategies.

## Abstract (web summary)

Longitudinal deep lipidome profiling reveals >800 lipid species, many of which are associated with health-to-disease transitions in diabetes, ageing and inflammation.

## Main

Lipids are an important and highly diverse class of molecules that have critical roles in cell structure, cell signalling and bioenergetics. Despite their critical roles in many biological processes, there is much to be learned about the diversity of lipids in humans, how their composition differs across people, and how they change over time at an individual level and during disease. Such information is expected to provide insights into biological processes such as ageing as well as the possible roles of lipids in health and disease.

High-throughput omics technologies provide new avenues to understand the molecular landscape of human physiology and its dynamic changes during health and disease. To date, many studies have used next-generation sequencing owing to its accessibility and cost-effectiveness^1^. Recently, mass spectrometry (MS) strategies have provided quantitative insights into the proteome^2,3^ at scale and depth. Metabolites, which can also be investigated using MS, have been studied to a lesser extent given their complex chemical diversity^4,5^. Lipids comprise a major, heterogeneous family of biomolecules within the metabolome and remain challenging to characterize owing to their wide range of physicochemical properties^6^ and the relatively small number of lipidomics studies.

Complex lipids can be divided into several classes and subclasses that are distinguished by lipid head groups and linkages to different aliphatic chains^7^. Lipids such as triacylglycerols (TAGs), diacylglycerols (DAGs), phosphatidylcholines (PCs), phosphatidylethanolamines (PEs), ceramides (CERs), sphingomyelins (SMs) and cholesterol esters (CEs) each consist of a specific backbone architecture conjugated to various fatty acids (FAs). The attached FAs can vary in the number of unsaturated bonds and their positions within acyl chains; together with the backbone, FAs confer distinct physicochemical properties and physiological roles. Lipids carry out and regulate many key functions, including redox homoeostasis, energy storage, intracellular and extracellular signalling, induction and resolution of acute and chronic inflammation^8–10^, and maintenance of electrochemical gradients across subcellular compartments. Abnormal lipid profiles (dyslipidaemia) have been associated with a range of diseases, including metabolic syndrome, type 2 diabetes (T2D), cancer, nephropathy and cardiovascular and neurodegenerative diseases, and may result from a combination of factors such as genetic heterogeneity, lifestyle and, as recently shown, inflammation related to coronavirus disease 2019 infection^11–13^.

One of the key roles of lipids in maintaining metabolic homoeostasis is to mediate the induction and attenuation of inflammatory processes (for example, leukotriene, prostanoid and endocannabinoid signalling)^8,9,14^. Because of the various roles lipids have in maintaining homoeostasis in humans, different lipid species or classes may influence perturbations that induce acute inflammation (for example, respiratory viral infections (RVIs)), as well as the resolution of inflammation, metabolic diseases (for example, T2D) and physiological processes (for example, ageing) that have been associated with changes in the regulation of chronic inflammation. In light of the diverse roles of lipids, it is important to understand their quantitative differences among individuals and their dynamics across phenotypes to characterize their potential roles in health and disease.

Here, we characterize the lipidome dynamics in >100 human participants followed for up to 9 years, covering periods of health and disease, using an MS-based approach that allows a broad array of lipid types to be measured rapidly, quantitatively and rigorously^15,16^. We identified distinct longitudinal lipid signatures that link lipid profiles to the microbiome, ageing and different clinical pathophysiologies, including insulin resistance (IR) and chronic and acute inflammation. Our results provide valuable insights into the associations of key lipids and lipid subclasses with distinct metabolic health states in humans, and serve as a unique resource to the scientific community.

## Results

### Comprehensive lipid profiling of a longitudinal cohort

From a cohort of >100 participants with IR or insulin sensitivity (IS), we previously collected longitudinal molecular data comprising genome, transcriptome, proteome, metabolome and 16S microbiome data across different timepoints (~1,000 in total^17^). Within this cohort, we explored various molecular signatures in health and disease and identified hundreds of molecular pathways associated with metabolic, cardiovascular and oncologic pathophysiologies^17,18^. Here, we investigate the dynamics of a largely unexplored molecular layer—the ‘plasma lipidome’—and extend the longitudinal duration by 2 years to obtain a total of 1,539 samples.

**Fig. 1:**
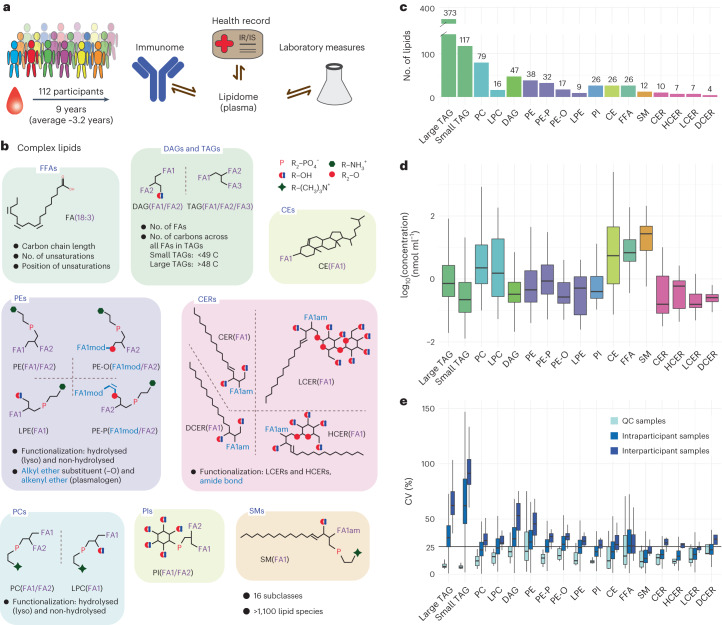
Longitudinal lipidomics profiling. **a**, Profiling, using >1,500 biosamples, across 112 participants followed for up to 9 years. Dynamic changes in the lipidome were characterized in the context of health status and medication history and in comparison with the participants’ cytokine, chemokine and metabolic profiles, as well as microbiome. **b**, Lipid subclasses investigated in this study. Lipid species, defined by a specific combination of backbone architecture and FAs, can be grouped based on their physicochemical properties. **c**, We analysed 846 lipids (*y* axis) across multiple subclasses. **d**, Across all 112 participants (median estimated concentration across all participant-specific samples), lipid species (846) spanned a dynamic range of more than four orders of magnitude, with distinct estimated concentration ranges for each lipid species and subclass. **e**, Comparison of the CVs of QC (*n* = 104), intraparticipant and interparticipant samples. All boxplots report the 25% (lower hinge), 50% (centre line) and 75% (upper hinge) quantiles. Whiskers indicate observations equal to or outside the hinge ± 1.5× the interquartile range (IQR). Outliers (beyond 1.5× the IQR) are not plotted. Source data

To investigate lipidome alterations associated with health, disease and lifestyle changes, plasma samples from 112 participants were profiled at a median of ten timepoints across 2–9 years (average 3.2 years; one participant was sampled 163 times across 9 years; Fig. 1a, Supplementary Data 1 and 2 and Supplementary Figs. 1 and 2). Samples were collected every 3 months when the participants were healthy and with an increased frequency of three to seven collections over 3 weeks during periods of illness (for example, RVI) or notable stress, as previously reported^17,18^. In addition to lipid profiling, we collected 50 clinical laboratory measurements at each sampling timepoint along with medical records (Supplementary Data 2). Finally, because samples were collected during periods of stress and illness, we also profiled 62 cytokines, chemokines and growth factors in plasma at the same timepoints.

The human lipidome was characterized using a high-throughput quantitative lipidomics pipeline (Lipidyzer) consisting of a triple-quadrupole mass spectrometer (Sciex QTRAP 5500) in conjunction with a differential mobility separation (DMS) device^15,16^. This setup allows the identification and robust quantification (estimated concentrations) of >1,000 lipid species across 16 subclasses (free FA (FFA), TAG, DAG, CE, PC, lysophosphatidylcholine (LPC), PE, alkyl ether substituent containing PE (PE-O), alkenyl ether (Plasmalogen) substituent containing PE (PE-P), lysophosphatidylethanolamine (LPE), SM, PI, CER, hexosylceramide (HCER), lactosylceramide (LCER) and dihydroceramide (DCER); Fig. 1b). In addition, we observed the differential behaviour of smaller and larger TAGs, which comprise ≤48 and ≥49 carbons across all FAs, respectively, and evaluated these separately in most analyses. For accurate quantification and to control for variance introduced during lipid extraction, we included a mix of 54 deuterated spike-in standards for nine lipid subclasses at known concentrations. Lipid species that were not present as labelled spike-in standards were normalized against the spike-in standards based on structural similarity and signal correlation (described in Methods).

We randomized the samples separately for lipid extraction and MS data acquisition. After filtering (described in Methods), we quantified, on average, 778 lipids in each sample and 846 lipid species across >1,600 samples (including quality control (QC) samples). We found the highest number (373) of lipid species in the large TAG subclass and the smallest number (4) in the DCER subclass (Fig. 1c). Lipids comprise chemically heterogeneous molecules that exert a broad spectrum of biological functions ranging from bioenergetics to cellular signalling. This is partially visible in lipid subclass-specific abundance distributions. Figure 1d shows the abundance distributions across more than four orders of magnitude and for each lipid subclass, and depicts two distinct properties: (1) the median abundance of that subclass and (2) the abundance range across all interrogated plasma samples (including healthy and disease timepoints). SMs and FFAs were observed, on average, as the most abundant subclasses, but they spanned a relatively small dynamic range. Other lipid subclasses, including LPCs, CEs and TAGs, had a lower median abundance but a much wider dynamic range.

**Extended Data Fig. 1:**
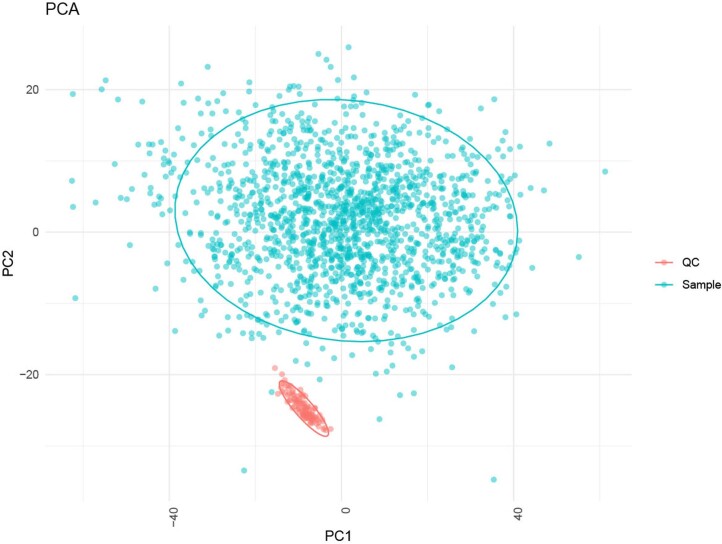
Principal component analysis for biosamples and quality controls. Principal component analysis of imputed log10 estimated nmol/ml concentrations calculated using prcomp (scaled and centered data). Quality control (red) compared to biosamples (light blue) show distinct clustering. Source data

Our study demonstrated high technical reproducibility. As anticipated, the 104 QC samples clustered distinctly (Extended Data Fig. 1); the median coefficient of variation (CV) for the QC samples was low, with values between 6.5% (small TAGs) and 20.7% (DAGs). In contrast, CVs calculated across participants and sampling timepoints ranged from 19.9% (SMs) to 91.4% (small TAGs), indicating sufficient assay reproducibility to discern biological differences. To ensure the highest robustness in our analysis, we focused on 736 lipid species for which (1) QC CVs were <20% and (2) CVs in biosamples were larger than CVs in QC samples. Except for FFAs, intraparticipant variance was consistently lower than interparticipant variance, suggesting that individual lipid signatures are distinct and stable over time (Fig. 1e). Interestingly, both small and large TAGs and ester- and ether-linked PEs (PE versus PE-O and PE-P) exhibited significant differences within their respective subclasses in terms of variance (Fig. 1e) and abundance distribution (Fig. 1d). This implies the existence of unique physiological and participant-specific differences, which may provide new insights into biological processes.

### Lipid signatures are highly individualized

We first sought to investigate lipid abundance differences across individuals by characterizing the lipidome in ‘healthy’ baseline samples, defined as samples from participants in the absence of any self-reported acute disease. This does not preclude latent, asymptomatic chronic conditions such as prediabetes or potential undiagnosed conditions. Overall, we analysed 802 healthy baseline samples derived from 96 participants from whom we collected samples at two or more timepoints. The number of baseline samples per participant is shown in Supplementary Fig. 3; most participants had approximately ten healthy visits, except one outlier with 52 healthy baseline samples.

**Fig. 2:**
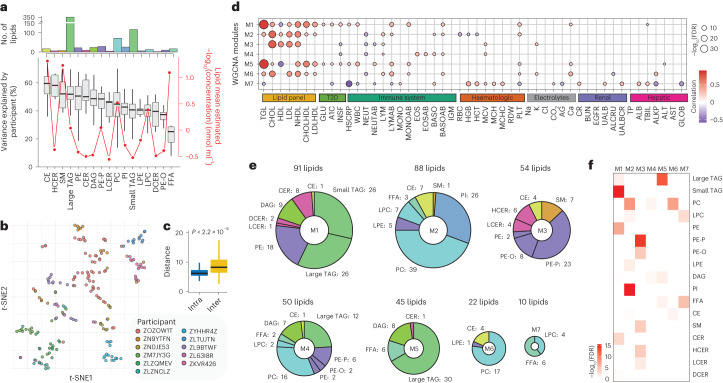
Interindividual differences in healthy baseline. **a**, Top, bar plot showing the number of lipid species per class ordered by the variance explained by the participant factor; bottom, boxplot showing the variance explained by participants in each lipid class (left *y* axis) and line graph showing the mean log_10_(estimated concentration) (red line, right *y* axis) of each lipid class. Variance decomposition analysis was conducted using *n* = 802 healthy samples. **b**, *t*-SNE clustering of 11 participants who contributed ≥12 healthy samples (*n* = 191), based on the 100 most personalized lipids. **c**, Intraparticipant distance, which refers to the Euclidean distance between each pair of samples belonging to the same participant, and interparticipant distance, which refers to the distance between the centroids from each pair of participants, for the *t*-SNE results. Boxplots report the 25% (lower hinge), 50% (centre line) and 75% (upper hinge) quantiles. Whiskers indicate observations equal to or outside the hinge ± 1.5× the IQR. Outliers (beyond 1.5× the IQR) are not plotted. The intraparticipant and interparticipant distances were compared using a two-sided *t* test. **d**, WGCNA modules and their correlation (BH-adjusted FDR cut-off of 5%) with clinical measures. Dot size depicts the BH-adjusted −log_10_(FDR). The colour scale indicates the degree and direction of the correlation. TGL, total triglyceride; CHOL, total cholesterol; NHDL, non-HDL; CHOLHDL, cholesterol to HDL ratio; LDLHDL, LDL to HDL ratio; GLU, glucose; INSF, fasting insulin; HSCRP, high-sensitivity CRP; WBC, white blood cell count; NEUT, neutrophil percent; NEUTAB, neutrophil absolute count; LYM, lymphocyte percent; LYMAB, lymphocyte absolute count; MONO, monocyte percent; MONOAB, monocyte absolute count; EOS, eosinophil percent; EOSAB, eosinophil absolute count; BASO, basophil percent; BASOAB, basophil absolute count; IGM, immunoglobulin M; RBC, red blood cell count; HGB, haemoglobin; HCT, haematocrit; MCV, mean corpuscular volume; MCH, mean corpuscular haemoglobin; MCHC, mean corpuscular haemoglobin concentration; RDW, red cell distribution width; PLT, platelet; AG, albumin to globulin ratio; CR, creatinine; BUN, blood urea nitrogen; EGFR, estimated glomerular filtration rate; UALB, urine albumin; ALCRU, aluminium to creatinine ratio, urine; UALBCR, urine albumin to creatinine ratio; TP, total protein; ALB, albumin; TBIL, total bilirubin; ALKP, alkaline phosphatase; ALT, alanine aminotransferase; AST, aspartate aminotransferase; GLOB, globulin. **e**, Module composition for the WGCNA analysis shown in **c**, coloured by lipid subclass. **f**, Enrichment analysis results based on Fisher’s exact test, depicting the BH-adjusted −log_10_(FDR) for enriched subclasses for each WGCNA module. Source data

In comparison with the transcriptome, proteome and general metabolome, lipid signatures can be highly personalized when assessed longitudinally^19^. To investigate the participant specificity of lipid profiles for healthy sampling timepoints at timescales of months to years, we examined which lipid subclasses show the largest interindividual differences and quantified how much of the variance observed for each lipid species can be attributed to interparticipant differences (Fig. 2a). Many lipids, in particular among TAGs, SMs, HCERs and CEs, showed a high degree of participant-specific variance, in some cases >50%. In contrast, FFAs were found to have relatively low participant-specific variation. To further illustrate participant specificity, we performed *t*-distributed stochastic neighbour embedding (*t*-SNE) on data from participants with >12 healthy visits, based on 100 lipids that we determined to be the most personalized (Fig. 2b). Most, but not all, samples clustered by individual participants (Fig. 2b,c), showing that some lipids can comprise personalized signatures even across years.

### Key lipids are associated with important clinical measures

Given the high variation in specific lipid classes among individuals, we next investigated the degree to which global lipidome profiles from healthy baseline samples are associated with clinical measures. We first grouped lipids into seven modules by applying weighted gene correlation network analysis (WGCNA) based on the similarity of lipid profiles and then associated these seven modules with 50 clinical measures while controlling for the covariates sex, age, ethnicity and body mass index (BMI; Fig. 2d–f and Supplementary Fig. 4). Controlling for these covariates allows investigation of the direct associations between the lipid modules and clinical measures by ruling out potentially confounding effects from sex, age and BMI. Modules M1 and M5, which were enriched for CER and PE, as well as small TAG (mainly M1) and large TAG (mainly M5), showed the strongest positive association with T2D measures, including glycated haemoglobin (A1C), fasting blood glucose and fasting insulin. Moreover, they showed a positive association with inflammatory markers, including high-sensitivity C-reactive protein (CRP) level and white blood cell count, and a negative association with high-density lipoprotein (HDL; ‘good cholesterol’) levels. Hence, lipids in M1 and M2 have negative health associations based on conventional clinical measures. In contrast, M7, which contained some FFAs and LPCs, correlated with lower CRP and A1C levels. M3, which was enriched for PE-P and PE-O, showed an association with higher levels of HDL and lower levels of fasting insulin, and, compared with the dominant T2D patterns in M1 and M5, demonstrated a signature that is generally considered healthier.

In addition, we investigated lipid–microbiome associations and observed mostly negatively correlated lipids, including several TAG species for the bacterial family Oscillospiraceae and (L)PE, PC and CE for Clostridiaceae (Supplementary Fig. 5 and Supplementary Note 1). These microorganisms are known to be abundant in the gut of participants with IS in this cohort^20^, suggesting a potentially beneficial role of *Clostridia* in host lipid metabolism. Finally, an outlier analysis identified participants with abnormally high or low lipid signatures, some of which we could correlate with underlying medical conditions such as hepatic steatosis (Supplementary Fig. 6 and Supplementary Note 2). Overall, this global analysis suggests that many lipid subclasses are associated with and potentially have a role (for example, proinflammatory, anti-inflammatory or metabolic role) in clinical conditions, or may serve as biomarkers to stratify health states.

### Global lipidome disruption in IR

As many clinical measures were associated with specific lipid subclasses, we next determined how the lipidome is influenced by the chronic metabolic disorder IR. IR commonly occurs in T2D and is a condition in which cells, mainly muscle cells and adipocytes, are unresponsive to insulin, leading to high glucose levels in the blood. IR is often associated with chronic inflammation as well as metabolic syndrome, including dyslipidaemia, and can lead to non-alcoholic fatty liver disease. Elucidating how the lipid network is perturbed in individuals with IR is important to better understand the molecular mechanisms and prognosis of metabolic disorders.

**Fig. 3:**
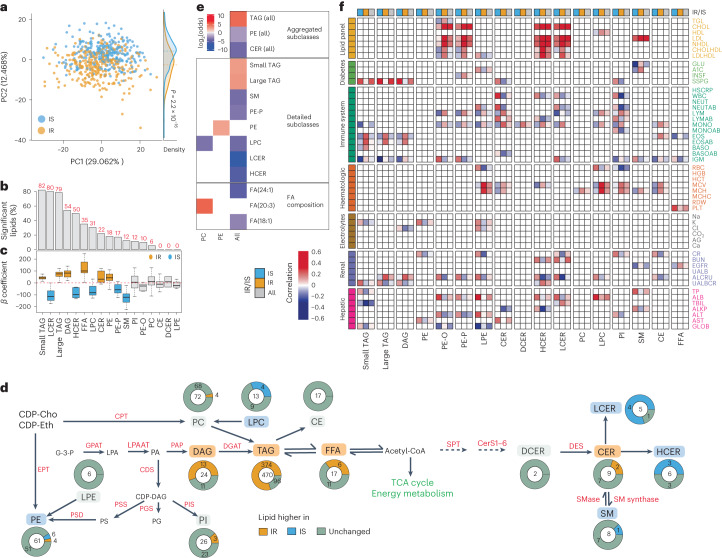
IR- and IS-associated lipid signatures. **a**, Principal component analysis comparing IR and IS. The density plot on the right indicates the distribution of eigenvectors for each data point along the second principal component (PC2). Eigenvector comparison between IR and IS was conducted using a two-sided *t* test. **b**, Regression analysis (*n* = 69): 424 of 736 lipids had significant correlations with SSPG (BH FDR < 5%; corrected for age, sex, ethnicity and baseline BMI). **c**, Boxplot depicting regression coefficients for the respective lipid classes by using 69 samples for which the SSPG level was measured at the visit. Larger coefficients indicate stronger associations with higher SSPG levels. Colour indicates distributions for which the 25th or 75th percentile is positive or negative. Boxplots report the 25% (lower hinge), 50% (centre line) and 75% (upper hinge) quantiles. Whiskers indicate observations equal to or outside the hinge ± 1.5× the IQR. Outliers (beyond 1.5× the IQR) are not plotted. **d**, Proportional differences between IR and IS detected in participants. Centre numbers indicate the total number of lipids in each class. Enzyme names are shown in red. CDP-Cho, cytidine diphosphocholine; CDP-Eth, cytidine diphosphoethanolamine; CPT, choline phosphotransferase; EPT, ethanolamine phosphotransferase; GPAT, glycerol-3-phosphate acyltransferase; LPAAT, lysophosphatidic acid acyltransferases; PAP, phosphatidate phosphatase; DGAT, DAG acyltransferase; G-3-P, glyceraldehyde-3-phosphate; CDS, CDP-diacylglycerol synthase; PSD, PS decarboxylase; PSS, PS synthase; PGS, PG synthase; PIS, PI synthase; SPT, serine palmitoyl transferase; CerS, ceramide synthase; SMase, sphingomyelinase; DES, dihydroceramide desaturase; Acetyl-CoA, acetyl coenzyme A; TCA, tricarboxylic acid. **e**, Enrichment analysis (Fisher’s exact test) performed on the coefficients from SSPG regression. Enriched annotations were calculated for positive coefficients with BH FDR < 10% (positive log_2_(odds)) and negative coefficients with BH FDR < 10% (negative log_2_(odds)). For enriched annotations, a BH FDR cut-off of 5% was applied. **f**, Correlations between clinical measures and lipid profiles for IR and IS. Correlations are shown when the correlations in IR and IS were significantly different and the absolute Δ correlations in IR and IS were >0.2. In addition, the overall correlations between lipids and clinical measures across IR and IS are depicted when the aforementioned two criteria were met. Source data

IR can be diagnosed by measuring the steady-state plasma glucose (SSPG) level after endogenous insulin secretion is suppressed and insulin and glucose are infused at fixed concentrations^21^. IR or IS (IR/IS) status was measured using SSPG assays in 69 participants, of whom 36 and 33 were classified as having IR (SSPG >150 mg dl^−1^) and IS (SSPG ≤150 mg dl^−1^), respectively. At the global level, we observed some capacity of lipid signatures to distinguish IR and IS (Fig. 3a). Using regression analyses that controlled for age, sex, ethnicity and baseline BMI, we resolved comprehensive differences between IR and IS across most lipid subclasses (Fig. 3b–d), such that more than half of the lipids (424) were significantly associated with SSPG levels. Lipids and lipid subclasses that had a significant positive correlation with SSPG included TAGs and DAGs, which is consistent with our observations (Fig. 2d) and previous reports of higher levels of these lipids in individuals with dyslipidaemia and metabolic syndrome^22,23^. We also observed subsets of CERs to have increased abundance, contributing to the development of obesity-induced IR in mice and humans^24^ (Fig. 3b,c), and making possible the lipid-based differentiation of IR and IS (Fig. 3a and Supplementary Fig. 7).

To investigate over- and under-representations of specific subgroups of lipids, we performed an enrichment analysis on positive and negative model coefficients. As TAGs comprise the largest subclass of lipids in our data and could dominate the results, we performed enrichment analyses separately for each lipid subclass and across all lipids (Fig. 3e). Enrichments were evaluated at the subclass level (Fig. 1b) and for FA composition (global saturation level and specific FAs). Importantly, to our knowledge, new associations were found, including an association of ether-linked PE (PE-P)—in contrast to PE in general—with lower SSPG levels. Ether-linked PEs are involved in cell signalling and can act as antioxidants^25^. Together with increased levels of TAGs with higher SSPG levels, reduced PE-P levels suggest IR-associated inflammation and may indicate a PE-mediated link between oxidative stress, inflammation and IR.

In Fig. 2d, we demonstrated lipid modules that correlate with a variety of clinical measures. As it is well documented that IR affects both lipid regulation (dyslipidaemia) and clinical phenotypes, we investigated whether associations between lipids and clinical measures are affected by the IR/IS status, which would have important health implications for these participants (Fig. 3f and Supplementary Fig. 8). Intriguingly, we found many significant differences in both the effect sizes and the direction of the correlation of lipid signatures and clinical measures between participants with IR and those with IS. For instance, in IR unlike in IS, the low-density lipoprotein (LDL) to HDL ratio was positively associated with the ether-linked PE-P and PE-O, and negatively associated with LPE (Fig. 3f). Moreover, in participants with IR and IS, we observed opposite correlations of immune and blood cell measurements with lipid subclasses, including A1C–SM, SSPG–CER and SSPG–PI, as well as immunoglobulin M–PE-P/PE-O, monocyte–PE-P/PE-O, eosinophil–TAG and white blood cell–PI (Fig. 3f). Overall, these data indicate that, depending on the IR/IS status, lipid–clinical measure associations can vary significantly and the key lipids involved in energy regulation, cell signalling and immune homoeostasis exhibit broad dysregulation in IR.

### Dynamic lipidome alterations during viral infections

In addition to their role in chronic inflammatory and metabolic conditions such as IR, complex lipids are key mediators of acute inflammatory responses, for example, by releasing arachidonic acid (FA(20:4)). Hence, complex lipids may be modified, released and activated during RVIs and possibly vaccinations while also having important roles in these processes in an IR-dependent manner.

**Fig. 4:**
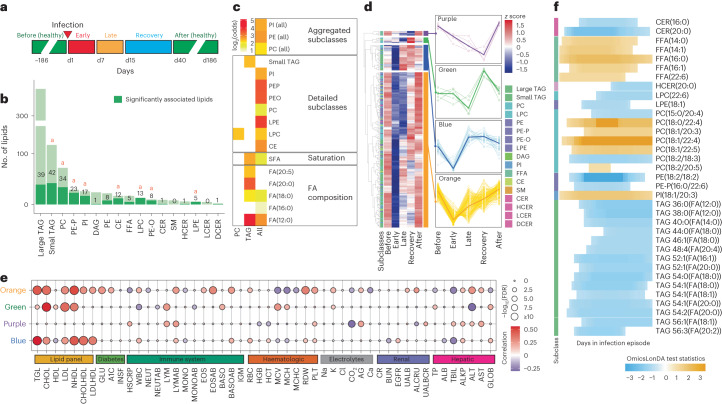
RVIs and vaccination. **a**, Longitudinal sampling at five timepoints during RVIs: before infection (healthy), early event, late event, recovery and after infection (healthy). **b**, Lipid class breakdown for all detected lipids. Dark green depicts 210 significantly changed lipids throughout RVI. ^a^Enriched subclass. Fisher’s exact test was used for the lipid class enrichment analysis of the significant lipids (BH FDR for each lipid subclass: CE, 3.35 × 10^−4^; CER, 0.95; DCER, 0.49; HCER, 0.87; LCER, 1; DAG, 1; FFA, 0.56; LPC, 6.32 × 10^−8^; LPE, 8.40 × 10^−3^; PC, 3.01 × 10^−4^; PE, 0.27; PE-O, 1.01 × 10^−3^; PE-P, 1.00 × 10^−8^; PI, 7.65 × 10^−5^; SM, 1; large TAG, 1; small TAG, 3.66 × 10^−2^). **c**, Lipid enrichment analysis for significantly changed lipids during RVI, across (left column) and within classes. **d**, Trajectory analysis of the 210 significantly changed lipids following RVI and their corresponding profiles in each cluster. **e**, Associations of lipid profiles in RVI and clinical measures. Depicted are correlations between the identified lipid clusters (**d**) and 50 clinical laboratory measures (BH FDR cut-off of 5%). Dot size depicts −log_10_(FDR); colour scale represents the correlation direction and degree. **f**, Differential profile of lipids that were significantly changed during RVI, comparing IR and IS. For each lipid feature, the shaded blocks demonstrate the time intervals during which the corresponding lipid was significantly different between IR and IS. The orange shaded blocks representing the lipid profiles at this time interval are dominant (with higher lipid levels) in IR, and the blue shaded blocks representing the lipid profiles at this time interval are dominant in IS. Source data

Participants in this cohort were densely sampled during periods of RVI (72 distinct RVI episodes in 36 participants for a total of 390 samples) and vaccination (44 episodes in 24 participants for a total of 275 visits; Supplementary Fig. 9). For both RVI and vaccination, we classified longitudinally collected samples as early-phase (days 1–6), later-phase (days 7–14) and recovery-phase (weeks 3–5) samples (Fig. 4a). Using linear models, we identified 210 lipids that were significantly changed during RVI (false discovery rate (FDR) < 10%) across most subclasses (Fig. 4b), some of which have previously been implicated in acute inflammation. For instance, PEs have been reported to have a critical role in apoptotic cell clearance and the aetiology of various viruses^26^. Another example is PIs, which bind to the respiratory syncytial virus with high affinity, preventing virus attachment to epithelial cells^27^. LPCs, which we observed in increased abundance during inflammation, have been demonstrated to have therapeutic effects (after intraperitoneal administration in mice) in severe infections through immune cell recruitment and modulation^28^.

To further investigate the lipid-associated processes that are involved in acute infection, we examined enriched lipids during infection (Fig. 4c). We observed significant changes in specific lipid subclasses, including ether-linked PEs and TAGs containing saturated FAs (SFAs) such as dodecanoic acid (FA(12:0)), following RVI. Dodecanoic acid and palmitic acid (FA(16:0)) are proinflammatory compounds that upregulate cyclooxygenase 2 (ref. ^29^) and have key roles in the activation of inflammatory responses. Overall, this suggests that different key lipid subclasses may be important for various aspects of viral biology as well as the immune response, and undergo significant changes during RVI.

To explore the choreography of lipid dynamics over time, we examined their trajectory during the different phases of RVI. The 210 significantly changed lipids were mapped to four major clusters, using a hierarchical clustering approach based on the Euclidean distance between lipid species as the similarity measure (Fig. 4d), and these main clusters were linked with clinical measures (Fig. 4e). Except for the green cluster, which was significantly enriched for PC, all profiles showed decreased levels during infection. The blue cluster was significantly enriched for small TAG and showed sharply decreased lipid levels in early RVI, with a rapid recovery that correlated with clinical measures of total lipids, including cholesterol and LDL. This indicates a metabolic shift in early infection, potentially to support increased energy metabolism. The orange cluster, enriched for LPC, large TAG and ether-linked PE, showed a similar profile to the blue cluster but a delayed recovery to baseline levels. Lipids in this cluster were positively correlated with the clinical lipid panel and blood glucose levels but negatively correlated with CRP level and neutrophils. This suggests that early changes in energy metabolism (reduction in lipid and blood glucose levels) are coupled with increased inflammation (reduction in LPC and ether-linked PE levels, as well as an increase in the CRP level and neutrophils) followed by a slow attenuation of inflammation at later stages of RVI. The purple cluster, which was enriched for FFA, represents slowly decreasing lipid levels and reached the lowest point during the RVI recovery phase before reverting to the baseline levels. In particular, the late-stage correlation with immune-related parameters (CRP level, lymphocyte count) suggests that reduction in the levels of some lipids in this cluster may relate to a temporary strong immunosuppression to attenuate early- to mid-phase inflammation and promote a return to homoeostasis. Overall, our data suggest links between differential responses of lipids and specific biological roles, with rapid shifts in energy metabolism to support inflammation early in infection and possible attenuation in later stages. Reflecting important global shifts in cell signalling, metabolism and inflammation during RVI, these lipids may allow the assessment of disease severity and prognosis or offer an opportunity for therapeutic intervention.

We next investigated whether individuals with IR and IS respond differently to infections and vaccination (Fig. 4f and Supplementary Fig. 10). Through a longitudinal differential analysis, we found distinct longitudinal profiles for IR and IS. We observed a higher abundance of several FFAs during the early stage of RVI and greater elevation of PC levels in the middle to late stage in participants with IR than in participants with IS. In contrast, TAGs and some PEs were differentially elevated in IS compared with IR throughout the middle to later stages of infection. The IR/IS-specific FFA and TAG responses may reflect the altered energy metabolism in IR, whereas differences in PCs and other lipid classes may indicate changes in immune-associated signalling pathways. Importantly, we found that the patterns after vaccination were distinct from those during infection (Supplementary Fig. 10). For example, fewer TAG species showed elevated levels in IS, whereas a distinct population of LCERs were upregulated in IR after vaccination. As individuals with T2D associated with IR often exert a more compromised immune response to RVI^17^, such changes may be biologically significant.

### Altered ageing of participants with IR

**Fig. 5:**
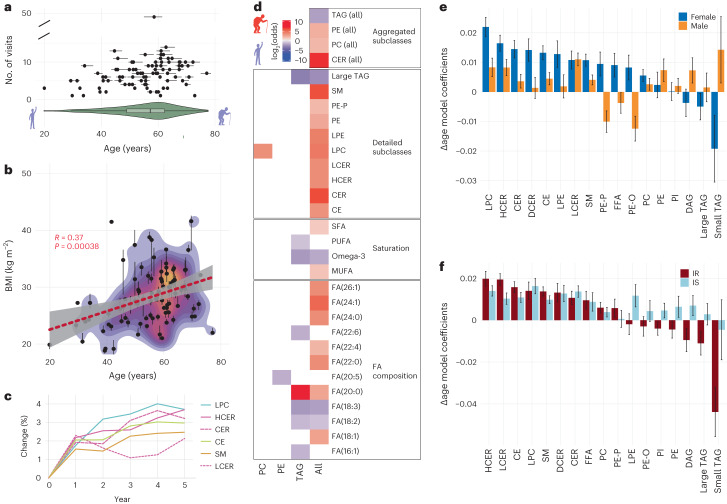
Age-associated changes in the lipidome. **a**, Median ages, age range (horizontal lines) and number of visits (*y* axis) of 90 healthy participants. Violin plot shows the distribution of age within the cohort. Inner boxplot reports the 25% (left hinge), 50% (centre line) and 75% (right hinge) quantiles. Whiskers indicate observations equal to or outside the hinge ± 1.5× the IQR. Outliers (beyond 1.5× the IQR) are not plotted. **b**, Correlation of median BMI and median age across healthy participants. Vertical lines depict the BMI range for each participant across all collected healthy samples. Regression line (red) from a linear model is shown with the 95% confidence band (grey). **c**, Significantly (BH FDR < 5%) changed lipid subclasses (percentage change for the summed untransformed concentration of respective lipid species) with ageing across 5 years based on the Δage model controlling for BMI and sample storage length. **d**, Fisher’s exact test enrichment analysis comparing physicochemical properties associated with higher age (positive log_2_(odds), red, determined for all positive Δage model coefficients at the lipid species level with a BH FDR of <10%) and those associated with lower age (negative log_2_(odds), blue, determined for all negative Δage model coefficients at the lipid species level with a BH FDR of <10%). Enrichments were calculated independently within lipid subclasses, as well as across all lipid species (‘all’). log_2_(odds) values are depicted for significant associations with lower or higher age (BH FDR < 5%). Infinite log_2_(odds) values are imputed with 0.5× the mean value of positive/negative log_2_(odds) determined across all data. MUFA, monounsaturated FA. **e**, Δage coefficients (ageing–sex) of individual lipid subclasses for male and female participants, controlling for sample storage length and BMI. **f**, Δage coefficients (ageing–IR/IS) of individual lipid subclasses for IR and IS, controlling for storage length, BMI and sex. For **e** and **f**, data are presented as the mean of estimated coefficients ± s.d., determined using an ordinary least-squares regression test. Source data

Ageing increases the risk of cardiovascular diseases and is accompanied by a variety of diseases including T2D^30,31^ and chronic inflammation^32^. In our study, the participants spanned an age range of 20–79 years (healthy timepoints, median 57 years) and were longitudinally sampled on average over 3 years (Fig. 5a). Across the cohort, we observed an increase in BMI with higher age (Fig. 5b). We previously identified age-associated molecular signatures in a subset of this cohort, including inflammation (acute-phase proteins), blood glucose and lipid metabolism (A1C, apolipoprotein A-IV protein), but had not investigated age-associated lipidome changes^33^. To identify lipids and pathways that change with ageing and may be associated with the development of age-related pathologies, such as chronic low-grade inflammation, we investigated longitudinal changes in the lipidome. In cross-sectional studies, lipid content can differ across participants with different ages, owing to biological ageing or the period during which the cohort aged, or other cohort effects. Periods and cohorts are social contexts affecting individuals and are inherently and mathematically confounded by the individuals’ age^34^. These comprise environmental factors differently affecting young and old participants, due to them being born in different generations, and include generation-dependent exposures that may also affect lipidome compositions (for example, diet, lifestyle and/or diseases) rather than actual age. However, we note that the longitudinal nature of our data better enabled us to eliminate some biases and focus on the same individual across time^34^. Furthermore, we previously did not observe major dietary changes in the cohort^18^. To identify lipid changes that occur with ageing in our longitudinal cohort, we used a linear model that estimates relative lipid changes as a function of the change in age (Δage model) while also controlling for sample storage length and BMI. With this model, we determined the ‘ageing’ effect (*β* coefficient) for each lipid subclass (Fig. 5c) and across lipid species (Fig. 5d).

We found that the levels of most lipid subclasses increased with ageing, most prominently CERs (LCER, HCER, DCER), SMs, LPCs and CEs, with some of the observed variance suggesting more complex lipid–ageing dependencies (Fig. 5c). A general increase in the levels of multiple lipid species and subclasses is consistent with previous observations^35,36^. Intriguingly, the levels of TAGs generally increased over time (Supplementary Fig. 11), but this trend disappeared when controlling for BMI. We performed an enrichment analysis on the Δage model coefficients at the species level and observed a shift in the physicochemical properties of lipids associated with ageing, including increased levels of SFAs and monounsaturated FAs, whereas the levels of polyunsaturated FAs (PUFAs) were reduced (Fig. 5d). This pattern has been previously associated with dyslipidaemia and inflammation^37^, underlining progressive deterioration of metabolic health during ageing. We also observed depleted levels of (beneficial) omega-3 FAs. In particular, the levels of docosahexaenoic acid (FA(22:6), TAG) and eicosapentaenoic acid (FA(20:5), PE) decreased with ageing. These omega-3 FAs have been indicated to have beneficial health effects by lowering plasma cholesterol levels and serving as precursors for mediators that resolve inflammation, such as resolvins, protectins and maresins^38,39^. In addition, decreased levels of linoleic acid (FA(18:2)) have been reported in aged skin^40^; our data show that this is also a significant ageing biomarker in blood plasma, suggesting a more systemic decrease. Through desaturation and elongation, linoleic acid is metabolized to arachidonic acid (FA(20:4)), which we found to increase in abundance with increasing age when we applied less stringent filtering (Supplementary Fig. 12), further substantiating a general shift towards inflammation with ageing. Furthermore, large and small TAGs showed distinct patterns, underlining the different functional roles along the TAG spectrum. Interestingly, the levels of LPCs, which have been implicated in cardiovascular diseases and neurodegeneration^41^ and some of which are anti-correlated with CRP (Fig. 2), increased with ageing, further underlining their pleiotropic role in human health. We also observed a strong sex dimorphism for multiple subclasses (Fig. 5e). Beyene et al. reported sex-associated differences in lyso- and ether-phospholipid metabolism^42^, which we confirmed in our study. In addition, we observed sex-associated differences for small TAGs as a prominent signature in ageing, with higher levels in men and lower levels in women.

We next investigated the extent to which IR alters molecular ageing signatures and observed that participants with IR had larger coefficients for multiple subclasses, including HCER, LCER, SM and CE, than participants with IS. Larger coefficients indicate that ageing-related changes may be accelerated in IR versus IS (controlling for storage length, sex and BMI; Fig. 5f). In contrast to previous reports that did not distinguish IR status^35^, our study identified a negative association between DAGs and ageing in participants with IR. Intriguingly, higher DAG levels are commonly linked to dyslipidaemia and IR^37,38^; however, similar to TAGs (see above), DAGs may have a stronger association with BMI, which was controlled for in the model. Moreover, PI and PE showed opposite ageing effects in participants with IR and IS, which suggests IR-specific changes in phospholipid metabolism with ageing. In sum, the composition of many lipid subclasses (that is, degree of unsaturation, omega-3 FAs, large TAGs, ether-linked PEs) changes significantly with ageing, a process that—for some lipid subclasses—differs between the sexes and is distinctly accelerated in the presence of IR.

### Specific associations of lipids with cytokines and chemokines

**Fig. 6:**
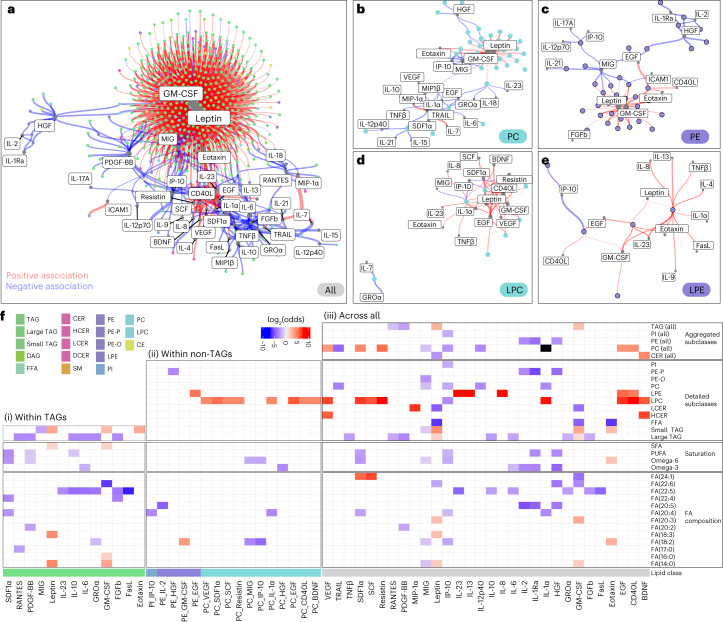
Lipid–cytokine associations. **a**–**e**, Network of 1,245 significant (BH FDR < 5%) lipid–cytokine associations, indicating positive (red) and negative (blue) associations calculated across 1,180 samples, across all lipids (**a**) and for PCs (**b**), PEs (**c**), LPCs (**d**) and LPEs (**e**). Networks were pruned based on a BH FDR of 5% for coefficients determined in linear mixed-effects models. Colour indicates lipid class; edge width represents coefficients; and node size represents node connectivity (popularity). The network was assembled using the ‘graphopt’ layout algorithm. **f**, Fisher’s exact test enrichment analysis comparing the physicochemical properties of lipids (*y* axis), at the subclass, global FA and individual FA level, that are associated with a particular cytokine (*x* axis). The analysis was performed for TAGs only (i), for all non-TAG lipids (ii) and across all lipids (iii). Enrichments (log_2_(odds)) among lipids with positive *β* coefficients (BH FDR < 10%) are indicated in red; enrichments (log_2_(odds)) among lipids with negative *β* coefficients (BH FDR < 10%) are indicated in blue; black denotes cases for which a certain property was enriched in both populations (positive and negative associations). log_2_(odds) values are depicted when the respective annotation was significantly associated with a BH FDR of <5%. Infinite log_2_(odds) values are imputed with 0.5× the positive/negative log_2_(odds) values determined across all data. IL-1Ra, IL-1 receptor antagonist; ICAM1, intercellular adhesion molecule 1; SDF1⍺, stromal cell-derived factor 1⍺; RANTES, regulated on activation, normal T cell expressed and secreted; PDGF-BB, platelet-derived growth factor-BB; GRO⍺, growth-regulated ⍺ protein; FasL, Fas ligand; TRAIL, tumour necrosis factor-related apoptosis-inducing ligand. Source data

Given the importance of cytokines, chemokines and growth factors in diverse biological processes, we characterized their relationship to lipids across homoeostasis and various pathophysiological disease processes in our longitudinal cohort. We investigated the degree to which the abundance of a particular lipid predicts the level of cytokines, chemokines or growth factors, controlling for BMI, sex, ethnicity and multiple measurements per participant as random effects across all samples and timepoints for which both measures were available (1,180 samples). Overall, we found 1,245 significant (FDR < 5%) positive and negative associations between a majority of lipids (580) and 40 cytokines, chemokines and growth factors (Fig. 6a).

The largest numbers of positive associations were between granulocyte–macrophage colony-stimulating factor (GM-CSF) and TAGs and between leptin and TAGs (Fig. 6a and Supplementary Fig. 13). The adipokine leptin regulates caloric intake and is commonly present in elevated levels in obesity, contributing to the associated inflammatory state^43^. Its amount in the blood correlates with the amount of adipose tissue. Its receptor is expressed in the hypothalamus, hippocampus and many immune cells; thus, it also acts as a neuroregulator and an immunoregulator^43,44^. The cytokine GM-CSF, originally defined as a haemopoietic growth factor, has other biological roles, including exerting proinflammatory effects^45–47^. These signatures are consistent with the inflammatory effect of the high TAG levels that we observed and are also found as a consequence of a high-fat diet, obesity and hepatic adiposity^48,49^. The pleiotropic cytokine interleukin-6 (IL-6), whose inflammatory and anti-inflammatory effects are context dependent^50^, together with the anti-inflammatory cytokine IL-10 (ref. ^51^), showed negative associations with some TAGs and clustered distinctly from the positive TAG–leptin and TAG–GM-CSF associations, suggesting functional differences among different TAG species in immunoregulatory networks (Fig. 6a). TAGs showed the overall highest number of associations with leptin and GM-CSF, whereas lipids from other subclasses, such as PE, PC and DAG, were also positively associated (Fig. 6b,c and Supplementary Fig. 13). In contrast, lyso species of PE and PC (Fig. 6d,e) showed fewer associations with and less central roles for GM-CSF and leptin. Overall, these results suggest regulatory commonalities across lipid classes (for example, positive associations of TAGs, DAGs, PCs and PEs with leptin) and differences within subclasses for proinflammatory and immunoregulatory pathways.

To elucidate the extent to which specific subsets of lipid species are associated with cytokines and chemokines, we performed an enrichment analysis (Fig. 6f). Overall, we observed strong associations of FAs with distinct cytokines. For instance, positive leptin–TAG associations were significantly enriched for SFA, the polyunsaturated FA(18:3) and small TAGs. In contrast, large TAGs were negatively associated with IL-6 and IL-10. Moreover, we observed a hub of negative associations between TAGs containing FA(22:5) and multiple cytokines, including the anti-inflammatory IL-10 and the proinflammatory IL-23, as well as IL-6. Enrichment of TAG subclasses for positive and negative associations within both proinflammatory and immunoregulatory cytokines suggests that TAG subclasses (in terms of both the length and saturation of the acyl chain) have distinct roles in immunoregulation and signalling.

In Fig. 2, we found that some LPCs were associated with anti-inflammatory, hence healthier, signatures. Here, LPCs were positively associated with several growth factors, such as epidermal growth factor (EGF), vascular endothelial growth factor (VEGF) and brain-derived neurotrophic factor (BDNF), and resistin. VEGF is involved in promoting angiogenesis, whereas BDNF and EGF promote cell proliferation, with BDNF having a cardinal role in neurogenesis and plasticity^52^. In addition, LPCs were positively associated with the soluble CD40 ligand (sCD40L), which is secreted by activated T cells and platelets during inflammation, as well as with the inflammatory cytokine IL-1⍺ and the adipose tissue-specific secretory factor resistin, which induces other cytokines and has been suggested to contribute to a chronic proinflammatory cascade in T2D^53^. Together, LPCs demonstrated contrasting associations, including some anti-inflammatory and tissue repair as well as proinflammatory signatures. These associations may highlight a difference between chronic inflammation (for example, mediated by factors such as resistin during T2D) and acute inflammation (for example, during an infection), which is strongly associated with high CRP levels. It may also reflect that both inflammatory and anti-inflammatory mediators are present in amounts that regulate a response so that it is effective but not excessive. Moreover, PCs containing linoleic acid (FA(18:2)) were negatively associated with the chemokines CXC motif ligand 9 (CXCL9; also known as MIG (monokine induced by interferon-γ (IFNγ))) and CXCL10 (also known as IP-10 (IFNγ-induced protein 10 kDa); Fig. 6b,f). CXCL9 and CXCL10 are induced by IFNγ to recruit cells to sites of inflammation; they bind to the same chemokine receptor, CXCR3. This association suggests that these lipids may affect immune cell migration during inflammation, in addition to their immune modulation role that we observed during RVI (Fig. 4). Overall, our multiomics data outline complex associations between cytokines and lipid subclasses as well as differential associations of lipids with specific FA compositions, suggesting distinct roles ranging from immune activation to immunosuppression.

## Discussion

Until recently, most omics studies have focused on transcriptomics, proteomics and, more recently, metabolomics, which is more closely associated with many phenotypes^54^. However, lipidomics remains largely underexplored despite lipids’ important roles in cell signalling, cell structure and energy management. The lipidome has been difficult to study owing to its complexity and the fact that it is derived from both endogenous and exogenous factors, such as the microbiome and lifestyle (diet and physical activity). Investigating the dynamic range of lipid changes, including which lipids change with acute or chronic conditions over what period, may reveal markers of early disease onset and progression as well as mechanistic insights that can be used to develop better and personalized treatments.

By following participants for up to 9 years, we identified highly participant-specific lipids and lipid subclasses (Figs. 1e and 2a–c), functional modules of lipids that map to clinical measures at baseline and throughout diseases (Figs. 2d,f and 3f), and lipid outlier signatures that may be predictive of diseases such as hepatic steatosis (Supplementary Fig. 6). Across perturbations such as IR (Fig. 3), viral infections (Fig. 4), ageing (Fig. 5) and cytokine–lipid associations (Fig. 6), we observed distinct behaviours among many lipid subclasses, such as ether- and ester-linked PEs, small and large TAGs, and lipids with specific FA configurations (for example, omega-3/omega-6 FAs, PUFAs and SFAs). Overall, our results point to the distinct biological roles of lipid subclasses and demonstrate that conventional clinical lipid profiles (that is, overall TAG levels) do not resolve many changes relevant to metabolic health.

Throughout our analyses, we consistently observed distinct behaviours between ester-linked and ether-linked PEs (PE versus PE-O/PE-P), as well as between two functionally distinct subgroups of TAGs (small TAGs (≤48 carbons across all FAs) and large TAGs (≥49 carbons across all FAs)). Ether-linked PEs have been implicated in cell signalling and as antioxidants^25^, and we found them to be significantly associated with healthy phenotypes including low SSPG levels and high HDL levels. In addition, ether-linked PEs are depleted early during infection, putatively increasing the inflammatory state, and/or depleted by scavenging radical oxygen species resulting from inflammation. In addition, PE, PE-P and PE-O show sex- and IR/IS-specific signatures during ageing (see below). Together, these observations suggest that ether-linked PEs are associated with health and chronically low levels of these PEs may have detrimental effects in humans. In this context, it will also be interesting to investigate other ether-linked lipid subclasses known to be detectable in blood plasma, such as PCs with alkyl ether substituent (PC-O) and PCs with alkenyl ether (Plasmalogen) substituent (PC-P), in future studies.

Recently, we reported the differential regulation of small and large TAGs within a 60-min recovery phase after exercise^19^. Here, we confirm and expand our previous observations suggesting new clinically relevant physiological roles of these TAGs within a much larger longitudinal cohort. Our data demonstrate that (1) many biological variations are captured in TAGs’ abundance profiles (Figs. 1e and 2a), which (2) are distinct for TAG subgroups including large and small TAGs, and those containing specific FAs (Figs. 5d and 6f). For instance, small TAGs show distinct associations with certain cytokines and chemokines and are rapidly depleted during early RVI, followed by a rapid recovery to baseline levels. Depletion of small TAGs during infection suggests an important role in energy metabolism and signalling to support early inflammation. In addition, during ageing, large and small TAGs differ markedly, suggesting distinct roles in ageing-related energy metabolism and lipid-mediated signalling. TAGs also showed the highest technical reproducibility in this study, making them an ideal target for new biomarkers at the subclass and species levels. We therefore propose that small and large TAGs as well as ether-linked PEs could be further explored as health biomarkers. Moreover, dietary supplements affecting plasma levels may provide a therapeutic avenue to reduce chronic inflammation and the detrimental effects of ageing and other conditions.

In addition to identifying lipids that decrease in abundance with ageing, such as PUFAs, omega-3 FAs, FA(18:3) and FA(18:2), we identified many lipid subclasses and properties that are enhanced with ageing, including the proinflammatory CEs, CERs, SMs, arachidonic acid and SFAs, as well as LPCs whose role could be more ambiguous. Intriguingly, some of these effects were stronger (for example, HCERs, CEs, SMs) or directionally different (for example, PEs, DAGs) in participants with IR, which can be interpreted as accelerated or differential ageing. IR is associated with chronic inflammation and can lead to dyslipidaemia and metabolic syndrome, which, in turn, increases the risk of age-related morbidities such as diabetes or cardiovascular diseases. Together with a distinct regulation between IR and IS, these observations indicate a large-scale realignment of chronic inflammatory processes during ageing, which may be accelerated in individuals with IR. Moreover, we observed strong sex-specific ageing signatures for lipid subclasses including CE. As cholesterol is a precursor of steroid hormones, many of these sex differences in CE levels may relate to or even cause differences in sex-specific hormone levels.

In addition to ageing-related differences, we found significant differences in both the effect sizes and the direction of the correlation of lipid subclasses and clinical measures between participants with IR and those with IS. Importantly, our models controlled for—among other variables—BMI, which is commonly associated with IR. Our analysis therefore highlights significant IR/IS differences separate from BMI effects. Key IR/IS-related differences include clinical markers related to T2D and the immune response; haematological, hepatic and renal measures; as well as electrolytes. For instance, PE-P/PE-O, LPE and LPC showed distinct directions of associations with multiple clinical measures, such as the LDL to HDL ratio and various cell populations, for IR and IS, expanding the previously suggested interplay between clinical measures and complex lipids^41^. As many lipids have both bioenergetic and signalling functions, our observation indicates important differences in cellular signalling in participants with IR.

Overall, these results underscore that the holistic assessment of metabolic health state is highly useful, and in some cases necessary, to improve the interpretation of conventional clinical measures such as the LDL to HDL ratio.

We identified distinct lipid subclasses and species that change in various disease states such as acute (RVI) and chronic (diabetes, ageing) inflammatory conditions. The role of lipids in immunity is only partially understood given the complexity of both the immune system and lipid metabolism. Our analysis covered myriad positive and negative cytokine–lipid associations, providing a valuable reference and resource for future studies. An important finding in this study is the strong positive associations of GM-CSF, leptin and the chemokine eotaxin (CCL11) with small TAG species (Fig. 6). As GM-CSF and leptin are involved in regulating and promoting inflammation, their strong associations with small TAGs underscore the central and distinct role these lipids may have in immunoregulation in comparison with groups of large TAGs.

Moreover, our data provide strong evidence for the pleiotropic role of LPCs, which are categorized as proinflammatory^41^. We found a strong positive association with proinflammatory signalling molecules, including IL-1⍺ and sCD40L, in addition to positive associations with growth factors such as BDNF and negative correlations with the proinflammatory markers CRP and SSPG. There may be feedback loops such that a response is balanced, leading to the production of both inflammatory and anti-inflammatory cytokines with different kinetics. Inflammatory responses must be proportionate to the stimulus so that they are effective but not excessive (leading to damage, for example, a cytokine storm). After the initial acute inflammation, the response needs to diminish and anti-inflammatory signals need to increase. This process needs to be tuned in both magnitude and kinetics, and associated lipids may have a role in these responses. Although this study was not designed to mechanistically resolve the role of LPCs and other lipid subclasses that we observed to be longitudinally associated with, for instance, viral infections, our data provide a resource for future studies and highlight putatively competing roles that may depend on physiological and pathological contexts, including comorbidities, age, BMI or IR/IS status.

Although the study cohort is ethnically diverse and sex balanced similar to the US population, there are some limitations. (1) There is a bias towards middle-aged and highly educated participants with a higher proportion of individuals living in northern California. (2) Some of the insights were generated based on small sample numbers (for example, outlier analysis; Supplementary Fig. 6). Therefore, not all observations and findings may be generalizable to a wider population that is subject to different lifestyles. Although we identified many signatures that have been observed in previous studies, supporting the validity of our findings, lifestyle differences can have an effect^19,55–57^ and future studies should be designed to confirm and extend our observations. (3) Our lipidomics pipeline targets >1,100 lipid species but does not always resolve the exact molecule identity (for example, position of a double bond in FAs). To expand the set of lipids investigated in this study, we included phosphatidylinositol (PI) as a new lipid class in multiple-reaction-monitoring transitions (MRMs) and observed multiple PI species to be significantly altered, for example, during RVIs. For this lipid class, however, no internal spike-in standards were included in the sample preparation, which limits the accuracy of the abundance information for all PIs. Moreover, not all putatively detectable blood plasma lipids, including PC-O and PC-P, were monitored. (4) The biphasic extraction method used here is an established and efficient procedure that is compatible with high-quality non-glass laboratory equipment. However, we have observed that FFAs have higher baseline levels when extracted with non-glass material, which can lead to an overestimation of the levels of certain FFAs. This ratio compression can reduce the sensitivity for detecting subtle changes between participants. (5) We used descriptive models (that is, we did not evaluate the predictive power by using cross-validation and hold-out data). Although we confirmed many previous findings and observed new lipid–phenotype associations, correlations are not proof of causation. In addition, our models controlled for multiple covariates (for example, sex and BMI), allowing for separation of effects, such as lipid–ageing versus lipid–BMI associations. Ageing is a complex process, and increasing BMI may be a default process for industrialized societies and lifestyles. Moreover, high BMI has been linked to inflammation. Thus, controlling for BMI may not always be desirable for understanding age- and inflammation-related changes in these cohorts. (6) The roles of complex lipids are diverse, and the physiological impacts of lipids with specific properties (for example, the fraction of complex lipids containing arachidonic acid) may not be due to direct effects.

Our study provides a longitudinal, in-depth analysis of intricate lipid–health relationships during acute and chronic inflammation, metabolic diseases and ageing. The multitude of lipid–phenotype connections we have revealed here serve as a valuable resource for biomarker discovery, a starting point to investigate disease mechanisms, and a basis to conceive therapeutic and preventive strategies. For instance, although many lipids undergo intestinal lipolysis driven by endogenous enzymes and microbial processes, some dietary lipids are absorbed directly. Our research suggests a variety of potential dietary interventions that could improve human health. Supplements such as ether-linked PEs may alleviate inflammation, providing effective treatment for chronic inflammation and facilitating recovery after RVI by minimizing damage related to reactive oxidative agents. Alongside adjusting the small TAG to large TAG ratio, these lipids could also be instrumental in addressing ageing- and IR-associated dyslipidaemia. Moreover, some lipid species and subclasses, including FFAs, ether-linked PEs, CEs or CERs, demonstrate sex-specific and ageing-related patterns. This prominent sex dimorphism suggests that sex- and age-specific interventions should be considered and might enhance therapeutic effects. It would also be worthwhile to assess the impact of genetic polymorphisms, particularly in relation to lipid species and subclasses exhibiting high variance, such as certain TAG, DAG and PE species, which may be relevant for personalized interventions. Together, future studies should explore how altering the exogenous lipid intake (for example, through diet) or targeting lipid conversion enzymes can affect both plasma lipid signatures and clinical phenotypes such as IR, acute and chronic inflammation, and molecular ageing.

## Methods

### Study design

Participants were enrolled as ‘healthy volunteers’ in the framework of the National Institutes of Health integrated Human Microbiome Project 2 (ref. ^17^). Inclusion and exclusion criteria were previously described in detail^18^. Participants provided informed written consent for the study under research study protocol 23602 approved by the Stanford University Institutional Review Board.

### Lipid extraction

Plasma samples were prepared and analysed in a randomized order. Plasma was thawed on ice, and lipids were extracted using a biphasic separation technique (ice-cold methanol, methyl *tert*-butyl ether and water). A 260-μl volume of methanol and 40 μl of a spike-in standard (cat. no. 5040156, Sciex) were added to 40 μl of plasma, and the mixture was vortexed for 20 s. Lipids were extracted by adding 1,000 μl of methyl *tert*-butyl ether and incubating the samples under agitation for 30 min at 4 °C. Phase separation was induced by adding 250 μl of ice-cold water, followed by vortexing for 1 min and centrifugation at 14,000*g* for 15 min at 4 °C. The upper phase containing the lipids was collected, dried down under nitrogen and stored at −20 °C in 200 μl of methanol. On the day of MS acquisition, lipids were dried down under nitrogen and reconstituted with 300 μl of 10 mM ammonium acetate in a 9:1 mixture of methanol and toluene.

### Lipidomics data acquisition

The QTRAP 5500 system (Sciex) equipped with a DMS device (Lipidyzer) was operated with a Shimadzu SIL30AC autosampler for targeted lipidomics, with a modified strategy to include additional lipid species in the acquisition. To ensure robustness of results, the Lipidyzer was cleaned and tuned after each batch (every 48 h; Supplementary Fig. 14). The tuning solution contained 40 μl of the SPLASH internal standard mix, 100 μl of the Sciex tuning mix, 100 μl of Lyso-tune mix (1 mg ml^−1^ 17:1 lysophosphatidylglycerol (LPG), 1 mg ml^−1^ 17:1 lysophosphatidylserine (LPS), 0.1 mg ml^−1^ 17:1 lysophosphatidylinositol (LPI), 10 μg ml^−1^ lysophosphatidic acid (LPA)) and 760 μl toluene–methanol (1:9) with 10 mM ammonium acetate.

For lipid extracts from 40 μl of plasma, three acquisition methods were used. The injection volumes were 42, 50 and 39 μl for methods 1, 2 and 3, respectively. The source temperature was set to 150 °C for all methods. Methods 1 and 3 were operated with DMS enabled and at a separation voltage of 3,700 V. Lipid classes were monitored as follows: method 1—PC (140), PE (119), PE-O (36), PE-P (61), LPC (26), LPE (26); method 2—CE (26), CER (12), DCER (12), HCER (12), LCER (12), FFA (26), TAG (519), DAG (59); method 3—SM (12), phosphatidic acid (PA; 77), LPA (12), phosphatidylglycerol (PG; 78), LPG (16), PI (77), LPI (16), phosphatidylserine (PS; 78), LPS (16). Each transition was acquired 20 times (see Supplementary Data 2 for the compensation voltage, Q1 and Q3 masses, and dwell times). Method 1, method 2 and the positive mode of method 3 (SM) contained transitions of the Lipidyzer original setup. Method 3 negative mode targets additional lipids.

### Raw data extraction and processing

Data acquisition was performed similarly to the processing of the Lipidyzer Workflow Manager. First, *.wiff files were converted to *.mzML files with MSConvert (v.3.0), setting ‘write index’ and ‘TPP compatibility’ to true. For each raw file, data extraction was performed in R. In brief, *.mzML files were imported with


openMSfile(FileAndPath, backend = “pwiz”)chromatograms(‘openMSfile_output)


using mzR (v.2.6.2). Next, all transitions with more than two zero intensities throughout the 20 repeated measurements were excluded (reported as ‘not available’). For all remaining transitions, the mean intensity was calculated (excluding zero-intensity recordings). Lipid species identities were matched based on the Q1 and Q3 masses and the corresponding scan index (the order in which MRMs were scheduled) in methods 1 (negative mode: PC, PE, LPC, LPE), 2 (negative mode: FFA; positive mode: TAG, CE, DAG, CER, DCER, HCER, LCER) and 3 (negative mode: LPG, PG, LPI, PI, LPS, PS, LPA, PA; positive mode: SM). Note that the transitions PG, PS, PA and their respective lyso forms were not considered for analysis. For lipids monitored in methods 1 and 2, as well as SM (method 3), internal standards from the Sciex Lipidyzer internal spike (LPISTDKIT-102b) were matched according to the Sciex Lipidyzer protocol. Individual concentrations were estimated based on the known abundance of the corresponding spike-ins. In brief, concentrations (‘actual concentration’) of all spike-in standards were retrieved from the ‘certificate of analysis’ of the internal spikes and converted to ‘nmol ml^−1^’. Lipidyzer assumes that, in plasma, nmol g^−1^ = nmol ml^−1^. Internal spike stocks of individual lipid classes were mixed, dried down and resuspended in a volume to adjust their respective stock concentration to the expected plasma levels (using the Lipidyzer Workflow Manager as a reference). The internal spike area measured by MS was compared with that of the respective endogenous lipids to approximate the absolute concentrations of the endogenous lipids. For complex lipids with two identical FAs, it was assumed that the measured signal from the fragment ions was at 2× intensity. Lipids belonging to the additional classes in method 3 had no corresponding standards and were normalized based on one of the other spiked-in lipids, as detailed in the next section.

The Lipidyzer resolves an individual FA as part of a TAG within each transition, a layer of information that we use to evaluate changes in FA compositions. For analyses that do not rely on the specific FA composition depicted in Fig. 3c, we aggregated TAGs to groups defined by summed FA carbons and the number of unsaturations, summing the untransformed concentrations of the corresponding TAGs.

### Normalization of lipid intensities in method 3

Spike-in standards (internal spikes) were not available at the time of lipid extraction for the lipids monitored here. Although this allows a relative comparison across samples given a reproducible workflow, we desired to leverage the information of the other internal spikes to further normalize for variances introduced across samples. To that end, we performed a correlation analysis by using QC samples derived from the same stocks that were measured across all batches. The lipid intensities in these samples are expected to be the same, and the ratios of internal lipids compared with internal spikes will be the same if the variation introduced by lipid extraction or MS analysis affects them in a similar manner. This setup allowed us to identify spike-in standards to normalize the additional lipids monitored in method 3 (PA, LPA, PG, LPG, PS, LPS, PI, LPI). For this normalization, we only considered internal spikes of the classes PC, PE, LPC and LPE, as those were also acquired in positive mode with DMS enabled.

We calculated the Pearson correlation coefficients for log_10_(intensities) comparing internal spikes and the new lipid species, and selected pairs according to the following hierarchy: (1) the highest correlating internal spike with at least 50% complete observations across QC samples was selected; (2) if a match could not be determined for a lipid species–internal spike pair, we selected the internal spike that showed the highest correlation with any other lipid of the same class; and (3) if both (1) and (2) did not select an internal spike, the highest correlating spike-in standard across all additional lipid classes was selected.

### Abundance estimation of lipids in method 3

For all original lipid classes, internal spikes of known concentrations allow the approximation of absolute abundances. As described above, the missing internal spikes for new lipids in method 3 do not allow direct inference of absolute abundances. Using a linear regression model based on all the known concentrations of lipids in the samples, we predicted the concentrations of the new lipids. As the normalized abundances for these lipids (method 3) are not based on labelled spike-in of the same molecular class and thus do not account for ionization efficiency differences, they provide an estimate of the absolute abundance range of the new classes. Importantly, this normalization does not affect the comparison of the relative abundance of the same lipid species across samples.

### Lipidomics data filtering

To ensure the accuracy and reliability of our analysis, we implemented several data filtering criteria. First, we excluded from the analysis biosamples with >25% missing data. Additionally, lipids with <10% valid values, as determined by the Lipidyzer reporting requirements, were also excluded. To further ensure high-quality quantitative results for the results presented in Figs. 2–6, we removed any lipid with a CV of >20% in QC samples and the few lipids for which the CV in QC samples was higher than the CV across the remaining biosamples. Furthermore, owing to limitations in separation by DMS, we did not include PAs in our analysis. We also excluded PS/LPS and PG/LPG from the analysis as they showed a significant number of missing values. PI(16:0/18:3) was removed from the dataset owing to its association with incorrect masses. Finally, QC_73 from batch 21 was removed owing to separate clustering compared with all other QC samples.

### Internal spike-in reassignment

Four internal spikes were not consistently quantified across the samples (missingness rate >5%) and were substituted with similar deuterated (d) standards belonging to the same class: dDAG(16:0/18:3) missing in 21% of the samples was substituted with dDAG(16:0/18:2); dDAG(16:0/20:5) missing in 12% was substituted with dDAG(16:0/20:4); dPE(18:0/22:5) missing in 44% was substituted with dPE(18:0/20:4); and dPE(18:0/20:5) missing in 8% was substituted with dPE(18:0/20:4).

### Data normalization

Lipids were normalized based on the internal spike-in standards (see above), similar to the standard Lipidyzer workflow that has been validated previously. Within an expanded method published by Su et al., additional MRMs can be used for isotope correction^58^. Here, we did not acquire all of the MRMs needed for isotope correction. Although the extent of correction depends on the abundance of interfering species and can significantly mask the signal of a targeted lipid species, Su et al. reported that no species was corrected by >6% and, outside of TAGs, no species was corrected by >3% (ref. ^58^). Cytokines were obtained in three separate batches. Data were log_2_ transformed and corrected for the effect of the batches using the ‘dbnorm’ (v.0.2.2) package^59^. The ComBat model (sva (v.3.38.0))^60^ showed the best performance and thus was considered in this study.

### Data imputation

#### Lipidomics data

**Extended Data Fig. 2:**
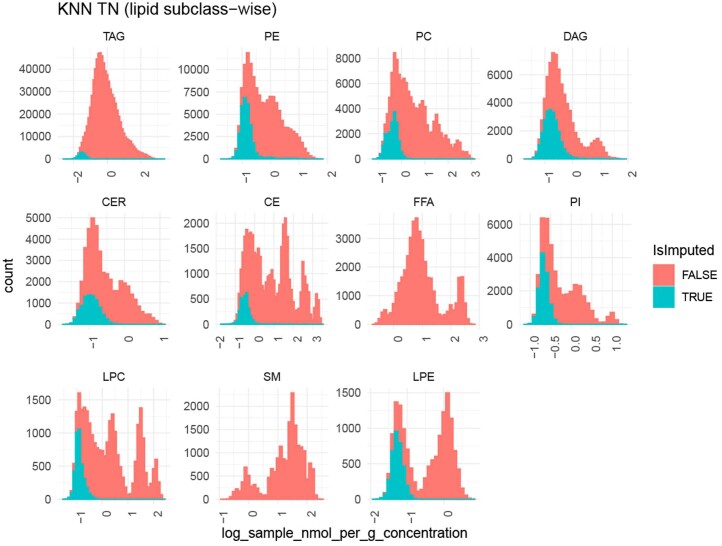
KNN-TN class-wise imputation. KNN-TN imputation of missing values (blue) for different lipid classes. Y-axis denotes counts, x axis denotes log10 estimated concentrations (nmol/ml). Source data

Missing values were imputed using a *K*-nearest-neighbour strategy that accounts for a truncated distribution (Extended Data Fig. 2)^61^. This approach involves drawing from intensities at the detection limit defined for each lipid class separately. This was a reasonable yet conservative assumption that allowed for the imputation of missing values without inflating fold changes by considering the sensitivity of MS. Missing weight measures in the ageing analysis were imputed by taking the mean between the two closest adjacent timepoints (Supplementary Data 2). Missing levels of several cytokines in batch 1 (that is, hepatocyte growth factor (HGF), basic fibroblast growth factor (FGFb), IL-8, IL-9, MIP-1⍺, stem cell factor (SCF) and tumour necrosis factor-β (TNFβ)) and batch 2 (that is, IFN⍺2 and FGFb) were imputed using the *K*-nearest-neighbour method with the number of neighbours being 10.

### Estimation of precision

CV was calculated for non-imputed, untransformed data. If, for a participant, multiple samples were collected on the same day, those were excluded. Only participants with at least three sampling timepoints were considered. Lipids with fewer than three quantifications were excluded. Note that, although the average intraparticipant CV was larger than the average QC CV, a small subset of low-abundance lipid species for a subset of participants showed lower CVs. These CVs emerged from the low signal that results in discrete quantifications from the mass analyser.

### Dimensionality reduction

*t*-SNE scatterplots were generated after log_2_ transformation and *z*-score scaling of the data using the R package ‘Rtsne’ (v.0.15) with the following parameters: perplexity = 5, *θ* = 0.5.

### WGCNA

Network analysis using self-reported healthy samples (Fig. 2d) was performed using the WGCNA R package (v.1.70-3). The soft-threshold power was optimized to achieve approximate scale-free topology (*R*^2^ > 0.8). Networks were constructed using the ‘blockwiseModules’ function. The network dendrogram was created using average linkage hierarchical clustering of the topological overlap dissimilarity matrix (1 − TOM). Modules were defined as branches of the dendrogram by using the hybrid dynamic tree-cutting method^62^, selecting a minimum module size of 5. Modules were presented by their first principal component (module eigengene) of the standardized expression profiles. Modules with eigengene correlations of >0.8 were merged together, generating seven lipid modules. Next, the Pearson correlation coefficients between the module eigengene and clinical measures were calculated using the ‘cor.test’ function in R (stats (v.3.6.2)), and all the obtained *P* values for the correlations were corrected for multiple hypotheses through the Benjamini–Hochberg (BH) procedure (stats (v.3.6.2)).

### Annotation enrichment analysis

To determine over- and under-representation of functional subgroups of lipids, we classified all lipid species based on their physicochemical properties (Supplementary Data 2) as reported in the LION database^63^. Under- or over-representation was evaluated using a hypergeometric test (Fisher’s exact test) or one-dimensional annotation enrichment^64,65^. Our dataset is dominated by TAG species, which could bias the enrichment analysis results. For that reason, we performed the enrichment analysis across all lipids and within subclasses. For each figure, the following statistics were used to calculate enrichments for categories: ‘OddEven_All’, ‘Omega_All’, ‘Saturation_All’, ‘Lipid_Class_Detailed_Special’ and ‘FA_All’, applying a BH FDR set to 0.1 by using the ‘p.adjust()’ function.

Figure 3e shows the Fisher’s exact test comparing positive SSPG coefficients with negative SSPG coefficients. Figure 4c shows the Fisher’s exact test determining whether the significantly changed lipids during infection (RVI) were enriched. Figure 5f shows the Fisher’s exact test comparing positive Δage coefficients with negative Δage coefficients. Figure 6f shows the Fisher’s exact test calculating enrichments in positive coefficients as well as negative coefficients. Negative infinite log_2_(odds) and positive infinite log_2_(odds) were imputed with 0.5× the minimum log_2_(odds) and 0.5× the maximum log_2_(odds), respectively. If a category was enriched among negative and positive coefficients for a lipid (that is, an over-representation was observed in the FDR-significant positive and negative coefficients), the enrichment was set to 0 and highlighted in black in the heat map.

### Correlation between lipids and clinical measures in IR/IS

Correlations between lipid levels and clinical measures were calculated using the ‘cor.test’ function in R. Lipidomics data and laboratory measures were both standardized before correlation calculation. To investigate differences between the correlations in IR and IS, we calculated the correlations by using only the healthy samples from participants with IR and IS, and we highlight only the correlation contrasts that were significantly different between IR and IS. The correlations between lipid levels and clinical measures from using all healthy samples from participants with IR and IS were also calculated and are presented as reference values.

### RVI clustering and correlation with clinical measures

*K*-means clustering was performed to investigate lipid similarities following infection by using the lipidomics data of infection events after log_2_ transformation and *z*-score scaling. We calculated the minimum centroid distance for a range of cluster numbers, and the optimal number was chosen using the ‘elbow’ method. The median values of the lipid profiles belonging to each cluster were correlated with the clinical measures to indicate medical implications. The correlations were calculated using the ‘cor.test’ function in R, and all the obtained *P* values for the correlations were corrected for multiple hypotheses using the BH procedure.

### RVI longitudinal IR and IS analysis

To detect the time intervals of differentially abundant lipids between IR and IS during infection events, we used a longitudinal analysis method, OmicsLonDA^66^. For each lipid in each group (IR or IS), we used a generalized additive mixed model for modelling nonlinear time-series abundance during the inflammation episodes. OmicsLonDA is an extension of MetaLonDA^67^ to account for correlated data, repeated measurements and multiple covariates. We accounted for sex, age, ethnicity and BMI as covariates, whereas participant identifiers were used as random effects. The *P* values for each lipid at each time interval (the time interval unit was set to 1 day) were obtained and then adjusted for multiple testing by using the BH procedure. We implemented this process on both infection and immunization events, identified the significantly different time intervals between IR and IS in both kinds of events, and compared these significant time intervals.

### Linear mixed models

#### SSPG association

To identify lipids that were associated with SSPG, linear mixed models were applied using log-transformed lipid measurements, controlling for participants, sex, ethnicity, age and BMI (Fig. 3). The R package ‘lme4’ (v.1.1-27.1) was used to construct the linear mixed models, as well as output estimates and nominal *P* values. The obtained raw *P* values were corrected for multiple hypotheses through the BH procedure by using the ‘p.adjust’ function in R.

#### Infection

To identify lipids that were significantly changed during infection episodes, linear mixed models were applied using log-transformed lipid measurements, controlling for participants, sex, ethnicity, age and BMI (Fig. 4). The R package ‘lme4’ (v.1.1-27.1) was used to construct the linear mixed models, as well as output estimates and nominal *P* values. The obtained raw *P* values were corrected for multiple hypotheses through the BH procedure by using the ‘p.adjust’ function in R.

#### Ageing

For each individual, ageing-associated lipid changes were calculated by subtracting measurements obtained at each visit from the baseline values (Fig. 5). Accordingly, the number of years since onset was calculated as the number of years from the first recorded measurement. To estimate the fractional changes in lipid measurements, we used a linear regression model with log-transformed lipid measurements, controlling for BMI and storage length (and sex if indicated in the figure). To control for potential biases related to the number of samples per individual, we excluded measurements from one participant with a uniquely large number of samples. To control for potential biases related to a few participants with measurements spread across a longer enrolment time, we excluded a few samples collected >5 years since onset. All coefficients and s.d. values were estimated using the ordinary least-squares implementation of the linear regression method in the ‘statsmodels’ package with the default parameters in Python (v.3.7). Linear models were run either at the lipid species level (Fig. 5d) or the lipid class level (sum of raw concentrations) for all participants (Fig. 5c), as well as for sex and IR/IS (Fig. 5e,f).

#### Cytokine–lipid association

We used linear mixed-effects models (lmer {lme4}) controlling for BMI, sex, ethnicity and participants (random effects) to estimate cytokine levels as a function of estimated lipid concentrations (Fig. 6). Both cytokine levels and lipid signals were scaled and centred (scale()). Restricted maximum likelihood was set to false, and *P* values were estimated using summ {jtools} and corrected for multiple-hypothesis testing with p.adjust() applying a BH FDR of <5% for network generation.

### Cytokine network

The cytokine–lipid network was constructed based on model coefficients by using the ‘graphopt’ layout algorithm in graphlayout{ggraph} (v.2.1.0), igraph (v.1.5.0) and tidygraph (v.1.2). The network was pruned to exclude all coefficients with a BH FDR of >0.05.

### Reporting summary

Further information on research design is available in the Nature Portfolio Reporting Summary linked to this article.

### Supplementary information

Supplementary Information Supplementary information on major findings (Supplementary Fig. 1), biosamples (Supplementary Figs. 2 and 3), weighted gene correlation network analysis (Supplementary Fig. 4), lipid–microbiome relation and outlier analysis (Supplementary Figs. 5 and 6), insulin resistance/insulin sensitivity analysis (Supplementary Figs. 7 and 8), association with infection and immunization (Supplementary Figs. 9 and 10), ageing (Supplementary Figs. 11 and 12), cytokine–lipid associations (Supplementary Fig. 13), mass spectrometry data processing scheme (Supplementary Fig. 14), lipid–microbiome associations (Supplementary Note 1) and lipid outlier analysis (Supplementary Note 2).

Reporting Summary

Supplementary Data 1 Processed lipid data.

Supplementary Data 2 Additional demographics, clinical data, microbiome–lipidome results, lipid annotations and mass spectrometry methods.

Supplementary Data 3 Source data for supplementary figures.

### Source data

Source Data Fig. 1 Global lipidome properties.

Source Data Fig. 2 Interindividual differences in healthy baseline.

Source Data Fig. 3 Insulin resistance- and insulin sensitivity-associated lipid signatures.

Source Data Fig. 4 Respiratory viral infections and vaccination.

Source Data Fig. 5 Age-associated changes in the lipidome.

Source Data Fig. 6 Lipid–cytokine associations.

Source Data Extended Data Fig. 1 Source data for principal component analysis comparing participant samples and quality control samples.

Source Data Extended Data Fig. 2 Source data for *K*-nearest-neighbour truncation imputation.

## Data Availability

Processed lipid data are provided as Supplementary Data 1. Raw mass spectrometry data are hosted on our portal at http://hmp2-data.stanford.edu/index.php under the substudy iPOP lipidomics as well as at https://www.metabolomicsworkbench.org/ under the direct link 10.21228/M8ZM5P. Cytokine and microbiome data are hosted at http://hmp2-data.stanford.edu/index.php. Lipids were classified partially based on their physicochemical properties reported in the LION database^63^. Source data are provided with this paper.

## Code availability

All software and algorithms are mentioned in the Methods section. Custom analysis scripts are hosted on the Stanford iPOP site (http://med.stanford.edu/ipop.html). Enrichment analysis was conducted using a modified version of the R AnnoCrawler^64^ package, with the corresponding lipid properties provided as Supplementary Data 2.

## References

[1] Adewale BA (2020). Will long-read sequencing technologies replace short-read sequencing technologies in the next 10 years?. Afr. J. Lab. Med..

[2] Aebersold R, Mann M (2016). Mass-spectrometric exploration of proteome structure and function. Nature.

[3] Ferdosi S (2022). Engineered nanoparticles enable deep proteomics studies at scale by leveraging tunable nano–bio interactions. Proc. Natl Acad. Sci. USA.

[4] Pinu FR, Goldansaz SA, Jaine J (2019). Translational metabolomics: current challenges and future opportunities. Metabolites.

[5] Newgard CB (2017). Metabolomics and metabolic diseases: where do we stand?. Cell Metab..

[6] Murphy RC (2018). Challenges in mass spectrometry-based lipidomics of neutral lipids. Trends Analyt. Chem..

[7] Fahy E (2009). Update of the LIPID MAPS comprehensive classification system for lipids. J. Lipid Res..

[8] Levy BD, Clish CB, Schmidt B, Gronert K, Serhan CN (2001). Lipid mediator class switching during acute inflammation: signals in resolution. Nat. Immunol..

[9] Serhan CN (2014). Pro-resolving lipid mediators are leads for resolution physiology. Nature.

[10] van Meer G, Voelker DR, Feigenson GW (2008). Membrane lipids: where they are and how they behave. Nat. Rev. Mol. Cell Biol..

[11] Shevchenko A, Simons K (2010). Lipidomics: coming to grips with lipid diversity. Nat. Rev. Mol. Cell Biol..

[12] Savelieff MG, Callaghan BC, Feldman EL (2020). The emerging role of dyslipidemia in diabetic microvascular complications. Curr. Opin. Endocrinol. Diabetes Obes..

[13] Caterino M (2021). Dysregulation of lipid metabolism and pathological inflammation in patients with COVID-19. Sci. Rep..

[14] Dennis EA, Norris PC (2015). Eicosanoid storm in infection and inflammation. Nat. Rev. Immunol..

[15] Contrepois K (2018). Cross-platform comparison of untargeted and targeted lipidomics approaches on aging mouse plasma. Sci. Rep..

[16] Ghorasaini M (2021). Cross-laboratory standardization of preclinical lipidomics using differential mobility spectrometry and multiple reaction monitoring. Anal. Chem..

[17] Zhou W (2019). Longitudinal multi-omics of host–microbe dynamics in prediabetes. Nature.

[18] Schüssler-Fiorenza Rose SM (2019). A longitudinal big data approach for precision health. Nat. Med..

[19] Contrepois K (2020). Molecular choreography of acute exercise. Cell.

[20] Zhou X (2020). Longitudinal analysis of serum cytokine levels and gut microbial abundance links IL-17/IL-22 with *Clostridia* and insulin sensitivity in humans. Diabetes.

[21] Hwu CM (2007). Surrogate estimates of insulin sensitivity in subjects with hypertension. J. Hum. Hypertens..

[22] Ginsberg HN, Zhang Y-L, Hernandez-Ono A (2005). Regulation of plasma triglycerides in insulin resistance and diabetes. Arch. Med. Res..

[23] Erion DM, Shulman GI (2010). Diacylglycerol-mediated insulin resistance. Nat. Med..

[24] Chaurasia B (2019). Targeting a ceramide double bond improves insulin resistance and hepatic steatosis. Science.

[25] Dean JM, Lodhi IJ (2018). Structural and functional roles of ether lipids. Protein Cell.

[26] Richard AS (2015). Virion-associated phosphatidylethanolamine promotes TIM1-mediated infection by Ebola, dengue, and West Nile viruses. Proc. Natl Acad. Sci. USA.

[27] Numata M (2015). Phosphatidylinositol inhibits respiratory syncytial virus infection. J. Lipid Res..

[28] Smani Y, Domínguez-Herrera J, Ibáñez-Martínez J, Pachón J (2015). Therapeutic efficacy of lysophosphatidylcholine in severe infections caused by *Acinetobacter baumannii*. Antimicrob. Agents Chemother..

[29] Lee JY, Sohn KH, Rhee SH, Hwang D (2001). Saturated fatty acids, but not unsaturated fatty acids, induce the expression of cyclooxygenase-2 mediated through Toll-like receptor 4. J. Biol. Chem..

[30] Jaul E, Barron J (2017). Age-related diseases and clinical and public health implications for the 85 years old and over population. Front. Public Health.

[31] Franceschi C (2018). The continuum of aging and age-related diseases: common mechanisms but different rates. Front. Med..

[32] Franceschi C, Campisi J (2014). Chronic inflammation (inflammaging) and its potential contribution to age-associated diseases. J. Gerontol. A Biol. Sci. Med. Sci..

[33] Ahadi S (2020). Personal aging markers and ageotypes revealed by deep longitudinal profiling. Nat. Med..

[34] Bell, A. & Jones, K. Age, period and cohort processes in longitudinal and life course analysis: a multilevel perspective. in *A Life Course Perspective on Health Trajectories and Transitions* (eds. Burton-Jeangros, C. et al.) (Springer, 2015); 10.1007/978-3-319-20484-0_10 27683926

[35] Slade E (2021). Age and sex are associated with the plasma lipidome: findings from the GOLDN study. Lipids Health Dis..

[36] Carrard J (2021). Metabolic view on human healthspan: a lipidome-wide association study. Metabolites.

[37] Klop B, Elte JWF, Cabezas MC (2013). Dyslipidemia in obesity: mechanisms and potential targets. Nutrients.

[38] Sears B, Perry M (2015). The role of fatty acids in insulin resistance. Lipids Health Dis..

[39] Sinclair HM (1984). Essential fatty acids in perspective. Hum. Nutr. Clin. Nutr..

[40] Kim EJ (2010). Skin aging and photoaging alter fatty acids composition, including 11,14,17-eicosatrienoic acid, in the epidermis of human skin. J. Korean Med. Sci..

[41] Law S-H (2019). An updated review of lysophosphatidylcholine metabolism in human diseases. Int. J. Mol. Sci..

[42] Beyene HB (2020). High-coverage plasma lipidomics reveals novel sex-specific lipidomic fingerprints of age and BMI: evidence from two large population cohort studies. PLoS Biol..

[43] Pérez-Pérez A (2017). Role of leptin as a link between metabolism and the immune system. Cytokine Growth Factor Rev..

[44] Abella V (2017). Leptin in the interplay of inflammation, metabolism and immune system disorders. Nat. Rev. Rheumatol..

[45] Bhattacharya P (2015). Dual role of GM-CSF as a pro-inflammatory and a regulatory cytokine: implications for immune therapy. J. Interferon Cytokine Res..

[46] Reed JA (2005). GM-CSF action in the CNS decreases food intake and body weight. J. Clin. Invest..

[47] Lee Y (2014). Anti-obesity effects of granulocyte-colony stimulating factor in Otsuka–Long–Evans–Tokushima fatty rats. PLoS ONE.

[48] Lee KMC, Achuthan AA, Hamilton JA (2020). GM-CSF: a promising target in inflammation and autoimmunity. Immunotargets Ther..

[49] Caro JF, Sinha MK, Kolaczynski JW, Zhang PL, Considine RV (1996). Leptin: the tale of an obesity gene. Diabetes.

[50] Tanaka T, Narazaki M, Kishimoto T (2018). Interleukin (IL-6) immunotherapy. Cold Spring Harb. Perspect. Biol..

[51] Pedersen BK (2013). Muscle as a secretory organ. Compr. Physiol..

[52] Numakawa T, Odaka H, Adachi N (2017). Actions of brain-derived neurotrophic factor and glucocorticoid stress in neurogenesis. Int. J. Mol. Sci..

[53] Siddiqui K, Joy SS, George TP (2020). Circulating resistin levels in relation with insulin resistance, inflammatory and endothelial dysfunction markers in patients with type 2 diabetes and impaired fasting glucose. Endocr. Metab. Sci..

[54] Fiehn O (2002). Metabolomics—the link between genotypes and phenotypes. Plant Mol. Biol..

[55] Lancaster SM (2022). Global, distinctive, and personal changes in molecular and microbial profiles by specific fibers in humans. Cell Host Microbe.

[56] Shen, X. et al. Multi-omics microsampling for the profiling of lifestyle-associated changes in health. *Nat. Biomed. Eng.*10.1038/s41551-022-00999-8 (2023). 10.1038/s41551-022-00999-8 PMC10805653 36658343

[57] Zhang X, Gao P, Snyder MP (2021). The exposome in the era of the quantified self. Annu. Rev. Biomed. Data Sci..

[58] Su B (2021). A DMS shotgun lipidomics workflow application to facilitate high-throughput, comprehensive lipidomics. J. Am. Soc. Mass Spectrom..

[59] Bararpour N (2021). DBnorm as an R package for the comparison and selection of appropriate statistical methods for batch effect correction in metabolomic studies. Sci. Rep..

[60] Johnson WE, Li C, Rabinovic A (2007). Adjusting batch effects in microarray expression data using empirical Bayes methods. Biostatistics.

[61] Shah JS (2017). Distribution based nearest neighbor imputation for truncated high dimensional data with applications to pre-clinical and clinical metabolomics studies. BMC Bioinformatics.

[62] Langfelder P, Zhang B, Horvath S (2008). Defining clusters from a hierarchical cluster tree: the Dynamic Tree Cut package for R. Bioinformatics.

[63] Molenaar MR (2019). LION/web: a web-based ontology enrichment tool for lipidomic data analysis. Gigascience.

[64] DansenCode. DansenCode/AnnoCrawler: AnnoCrawler. *Zenodo*10.5281/ZENODO.3939260 (2020).

[65] Cox J, Mann M (2012). 1D and 2D annotation enrichment: a statistical method integrating quantitative proteomics with complementary high-throughput data. BMC Bioinformatics.

[66] Metwally AA (2022). Robust identification of temporal biomarkers in longitudinal omics studies. Bioinformatics.

[67] Metwally AA (2018). MetaLonDA: a flexible R package for identifying time intervals of differentially abundant features in metagenomic longitudinal studies. Microbiome.

