# Supplementary material for: Dynamic lipidome alterations associated with human health, disease and ageing
Source: Nat Metab. 2023 Sep 11;5(9):1578–94. doi: 10.1038/s42255-023-00880-1 (PMC10513930; doi:10.1038/s42255-023-00880-1)
Supplement: Supplementary file 1 — Supplementary information on major findings (Supplementary Fig. 1), biosamples (Supplementary Figs. 2 and 3), weighted gene correlation network analysis (Supplementary Fig. 4), lipid–microbiome relation and outlier analysis (Supplementary Figs. 5 and 6), insulin resistance/insulin sensitivity analysis (Supplementary Figs. 7 and 8), association with infection and immunization (Supplementary Figs. 9 and 10), ageing (Supplementary Figs. 11 and 12), cytokine–lipid associations (Supplementary Fig. 13), mass spectrometry data processing scheme (Supplementary Fig. 14), lipid–microbiome associations (Supplementary Note 1) and lipid outlier analysis (Supplementary Note 2). [file 42255_2023_880_MOESM1_ESM.pdf]

nature metabolism

Article https://doi.org/10.1038/s42255-023-00880-1

Dynamic lipidome alterations associated with human health, disease and ageing

In the format provided by the authors and unedited

Lipidome Insights Across more than 1000 Biosamples

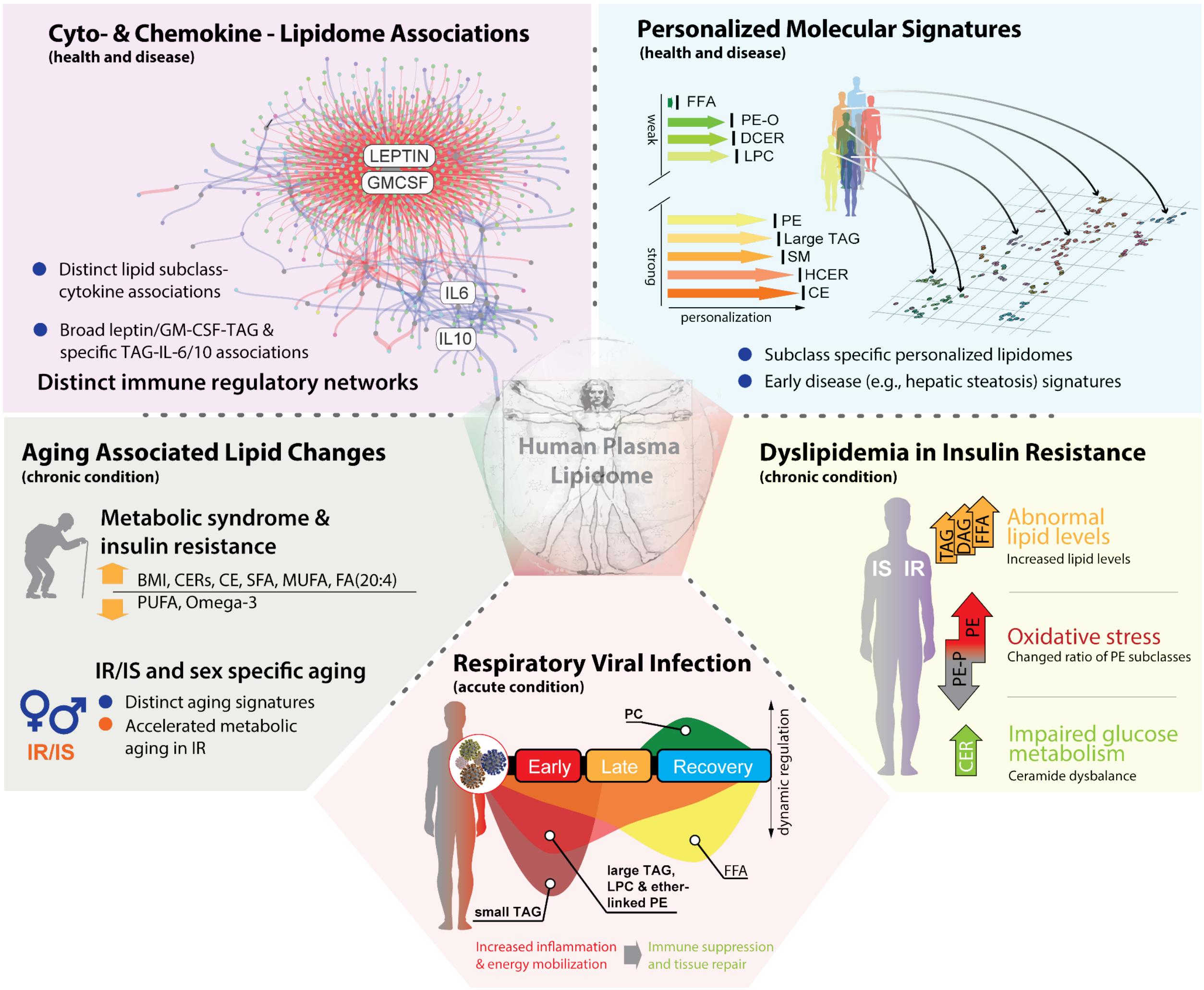

Supplementary Figure 1

The human plasma lipidome in health and disease

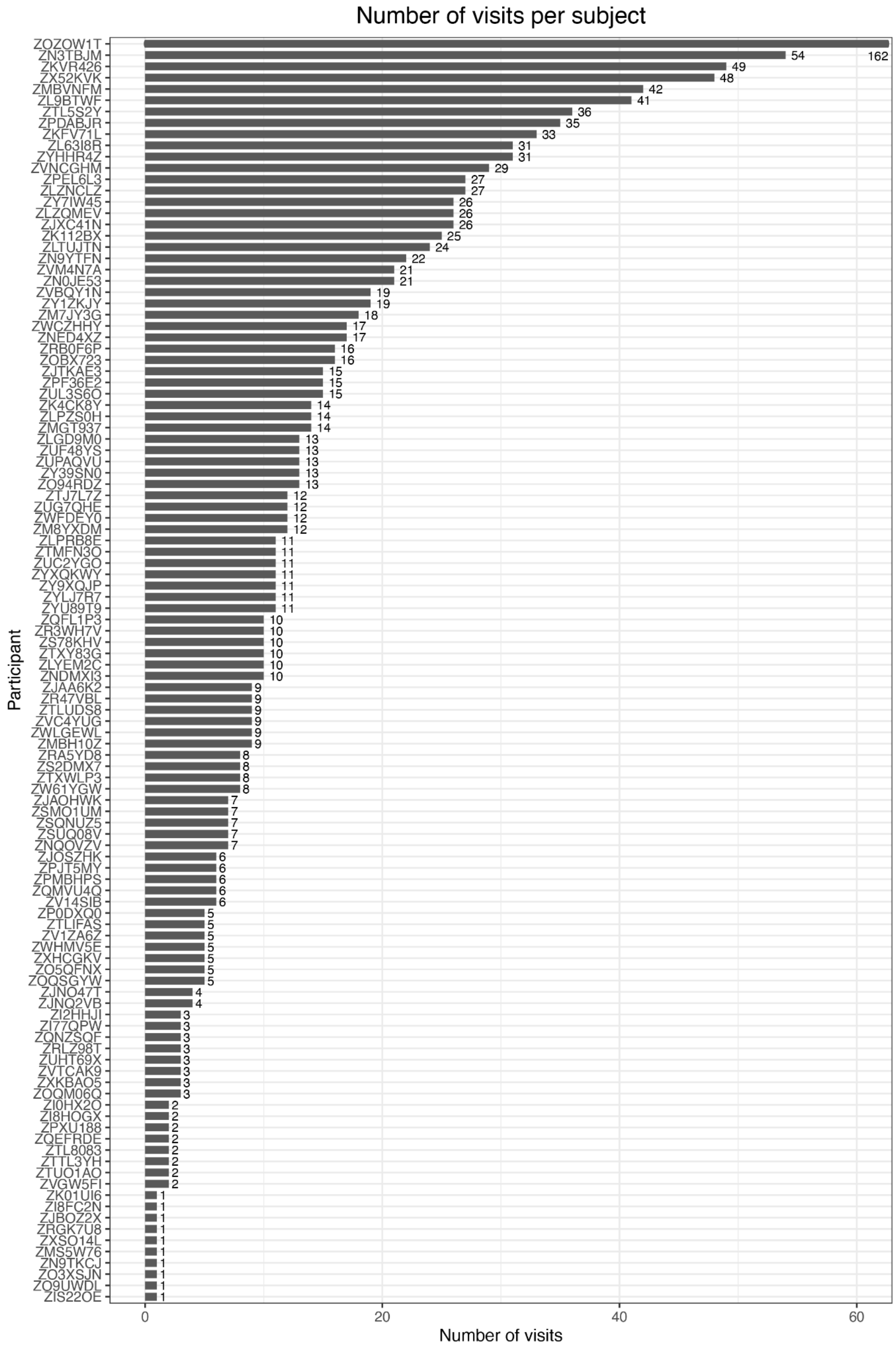

Supplementary Figure 2

Numbers of visits per subject in this study

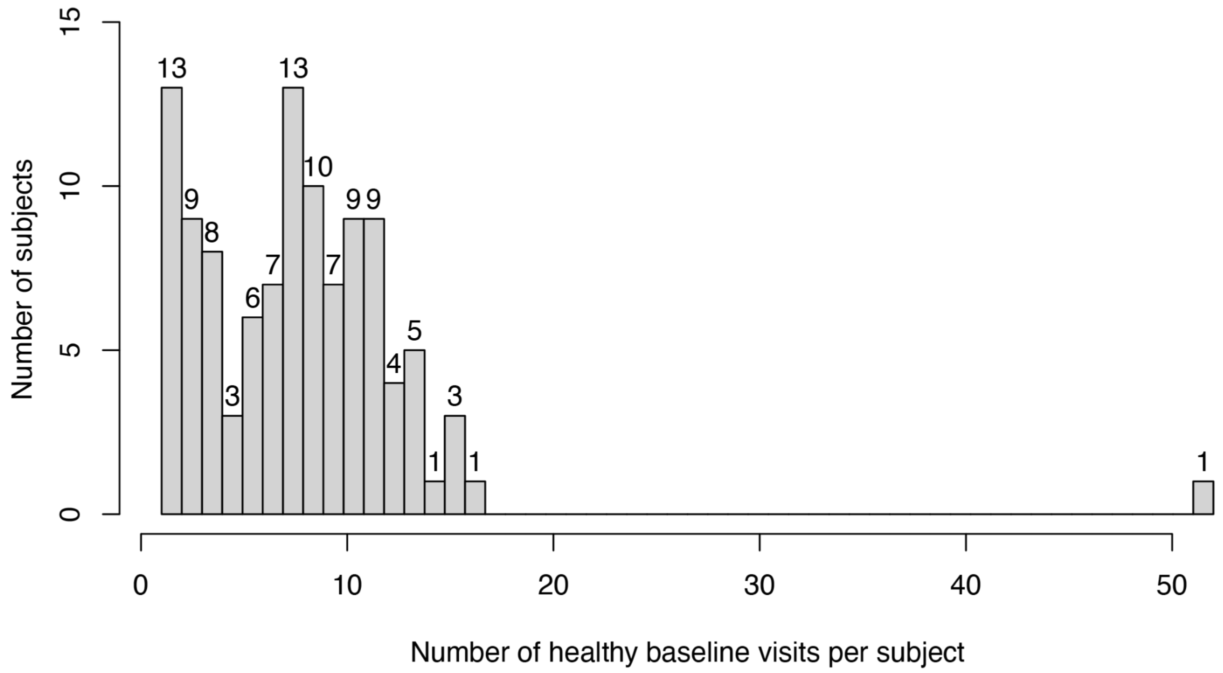

Supplementary Figure 3

Numbers of healthy baseline visits per subject

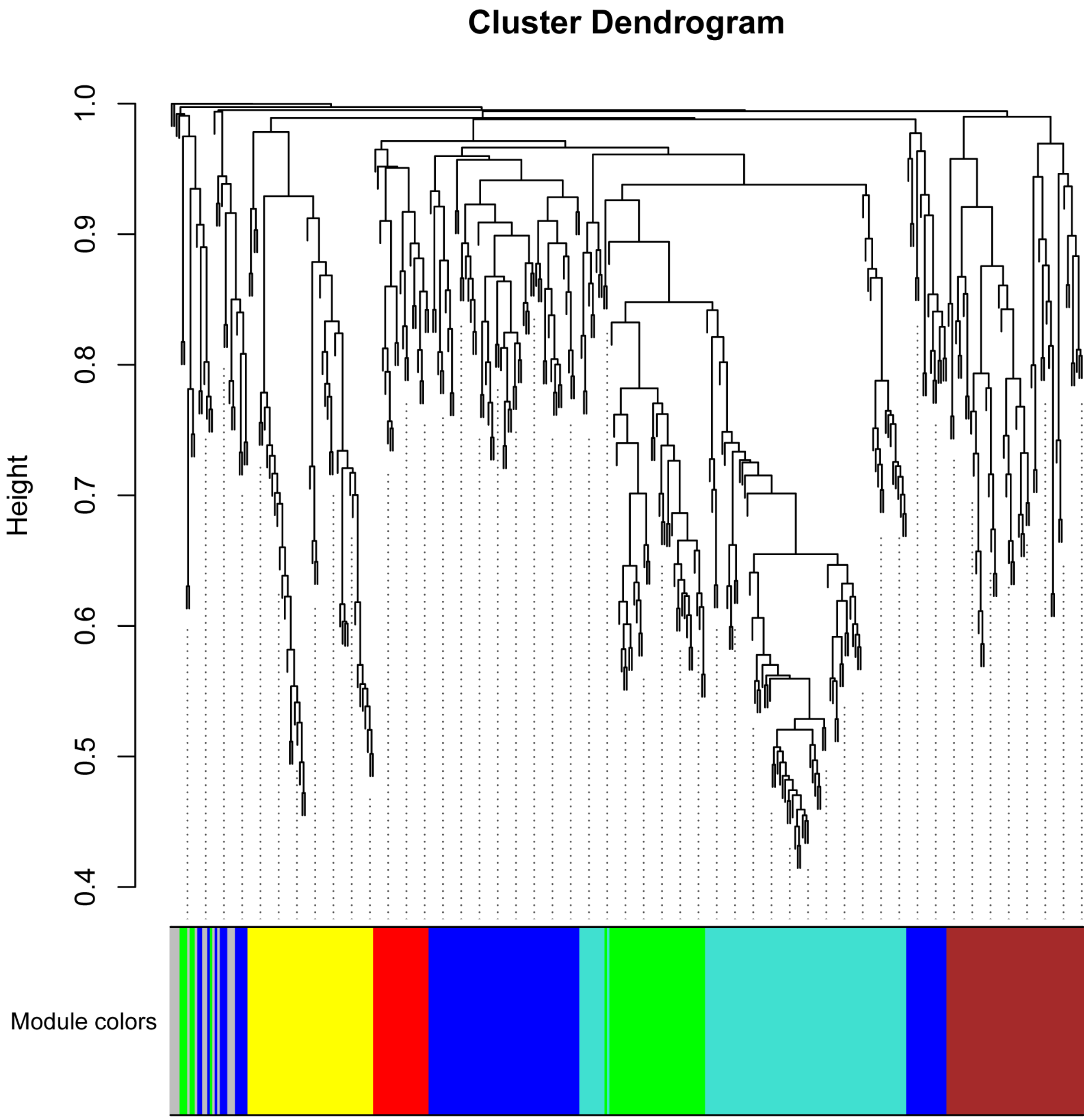

Supplementary Figure 4
WGCNA module visualization (Main Figure 2)

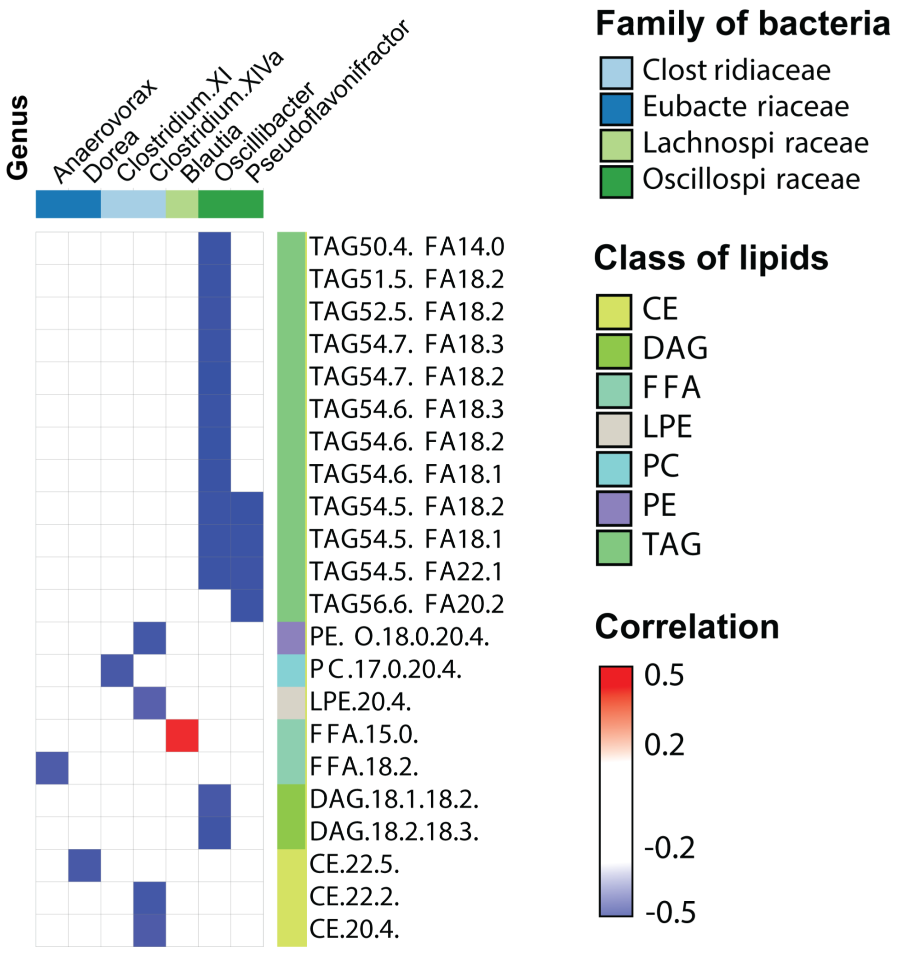

Supplementary Figure 5

The correlation between gut microbial genera and 846 lipid species are plotted. The pairs that are significantly correlated (p.adj. < 0.05) are color coded by its correlation coefficient. To adjust for multiple comparisons, a Benjamini & Hochberg (BH) correction was applied. Bacterial genera are color-annotated on the family level on column annotation, and lipid species are color-annotated as lipid class on row annotation. A two-sided Pearson correlation was used to determine the relationship between the mean value of microbial genera (proportion of reads belonging to this genera in relation to the log10 estimated nmol/ml abundance of lipids).

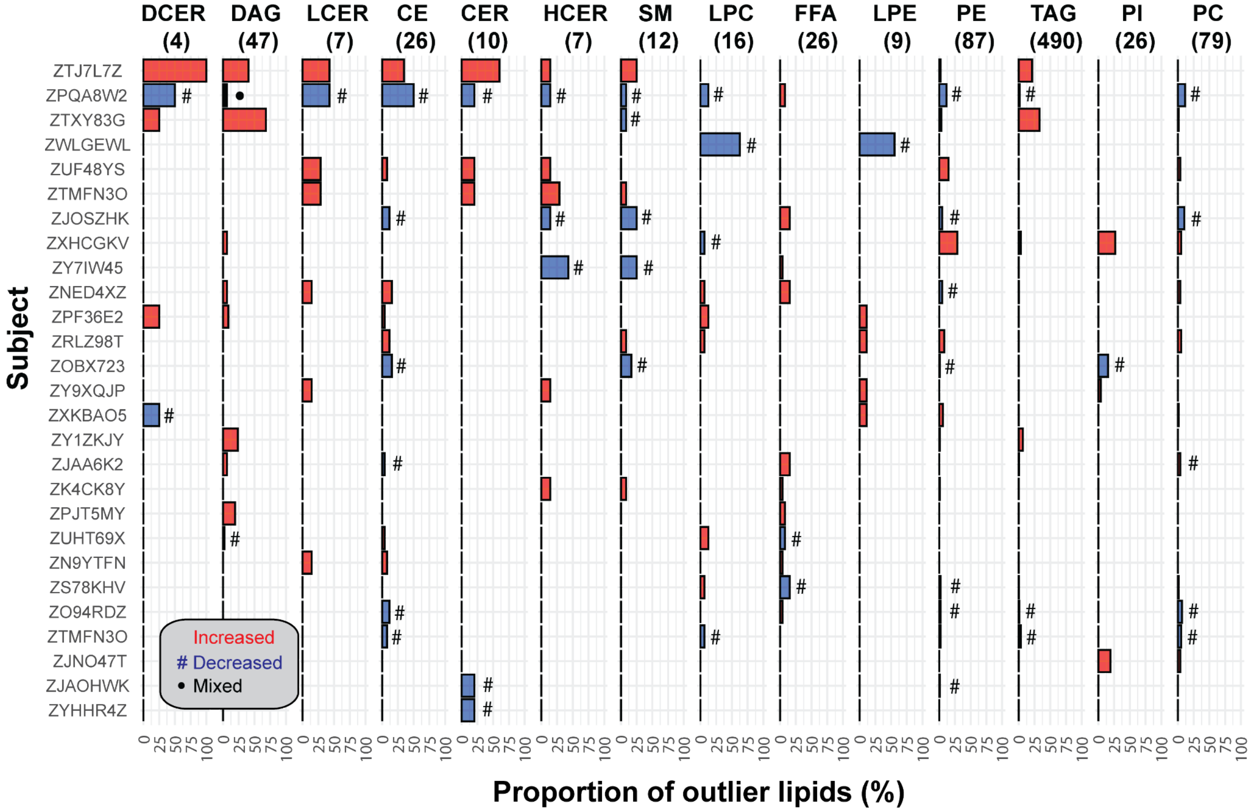

Supplementary Figure 6

Outlier analysis, depicting percent of significant lipid species outlier (calculated across 846 lipids species) per class and participant. Blue indicates upregulation, red depicts downregulation, gray shows mixed direction.

Effects of Insulin resstance on the lipidome

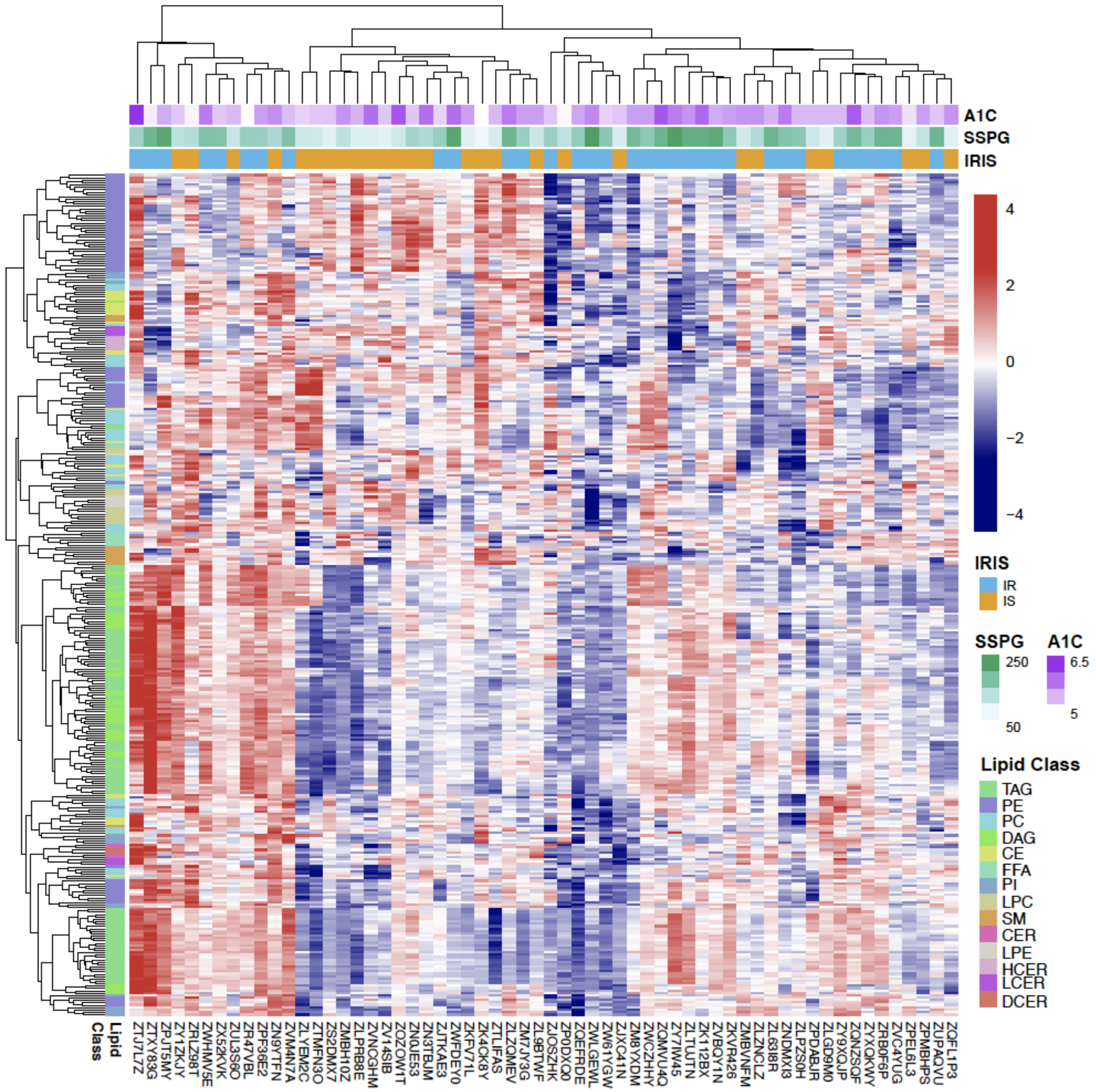

Supplementary Figure 7

Heatmap for the SSPG-associated lipids.

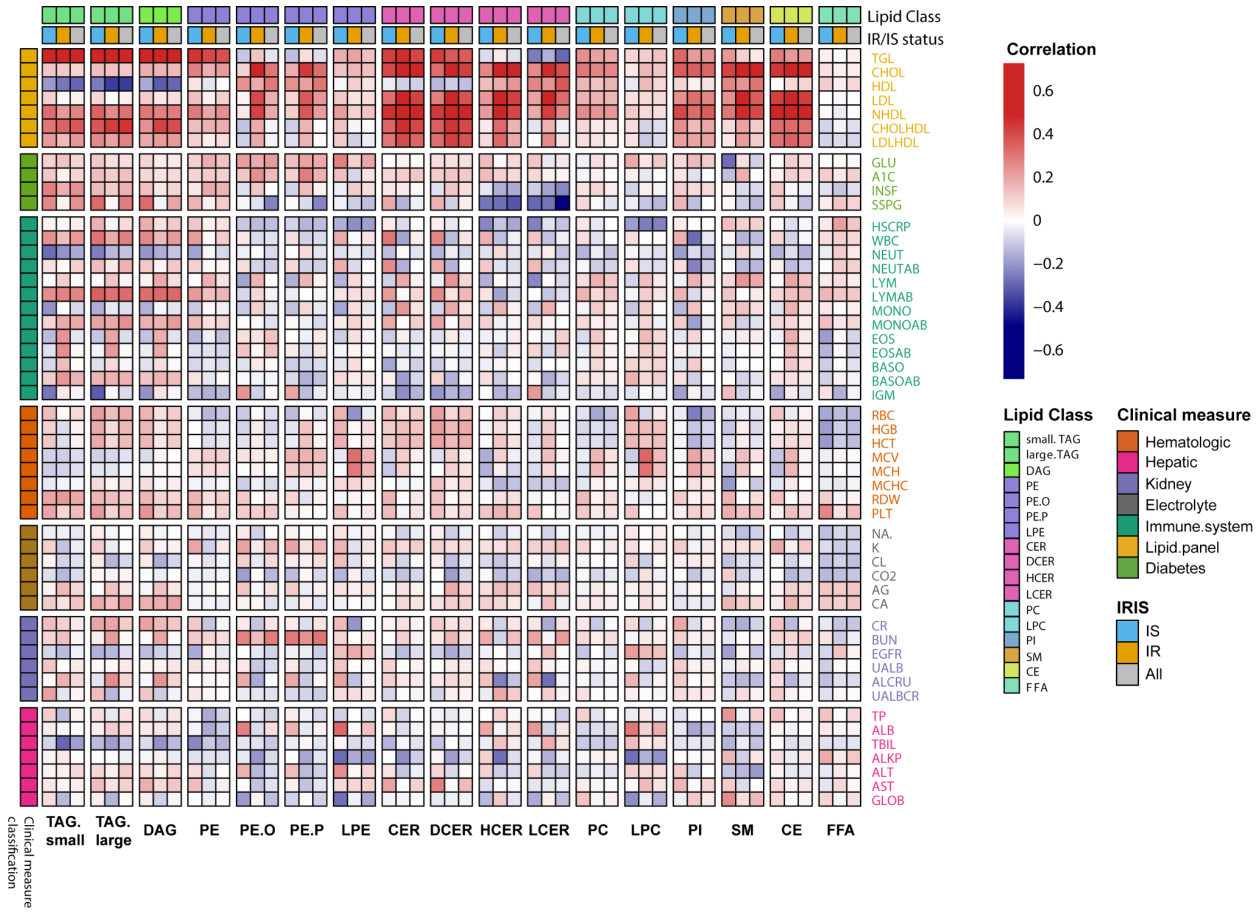

Supplementary Figure 8

Correlations between clinical measures with various categories and lipid profiles within IR and IS samples. In addition, the overall correlations between lipids and clinical measures across IR and IS, are depicted.

Lipid Changes During Infection and Immunization

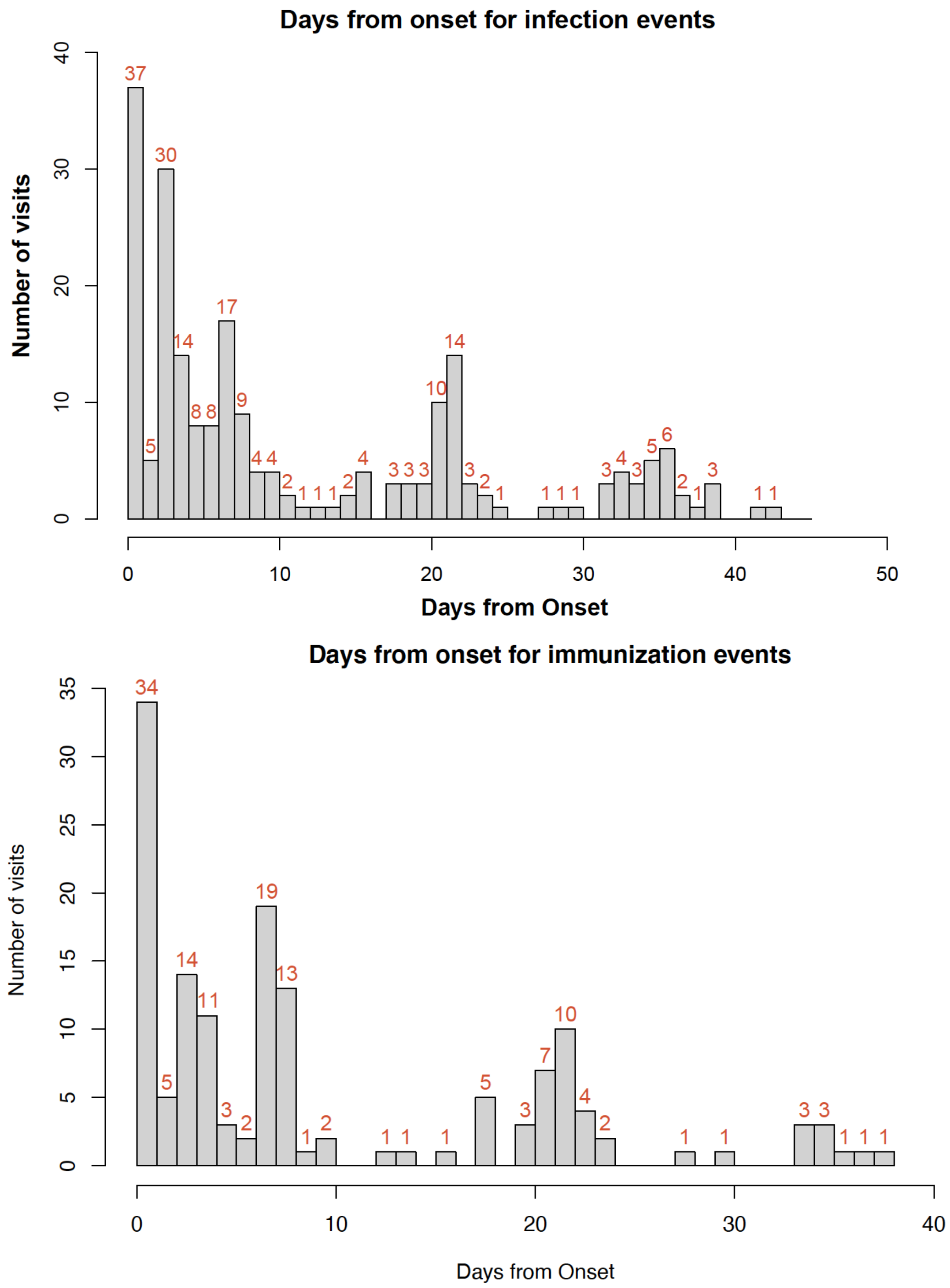

Supplementary Figure 9

Days from onset for infection and immunization adn associated visits.

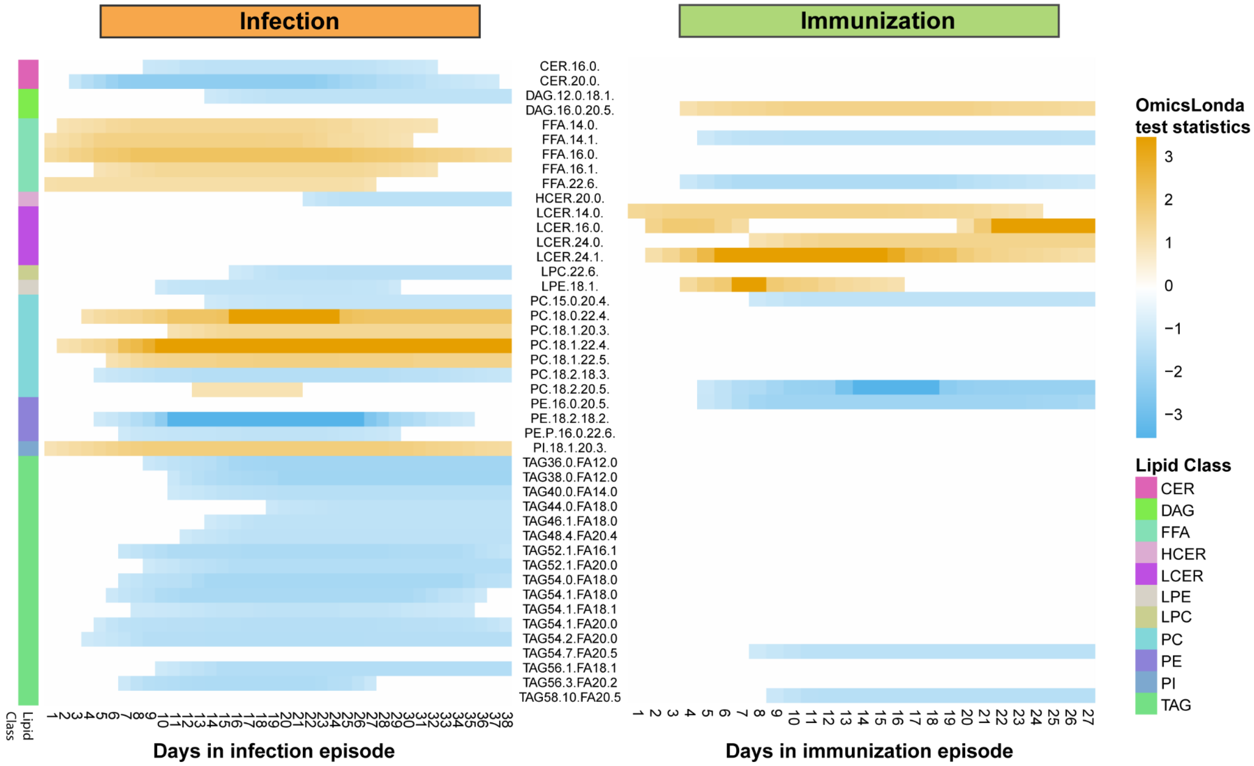

Supplementary Figure 10

Differential profile of lipids significantly changed during infection (left) and vaccination (right) comparing IR and IS. For each lipid feature, the shade blocks demonstrate the time intervals when the corresponding lipid is significantly different between IR and IS. The orange shade blocks representing the lipid profiles at this time interval are dominant (with higher lipid levels) in IR, and the blue shade blocks representing the lipid profiles at this time interval are dominant in IS.

Aging Associated Lipid Changes

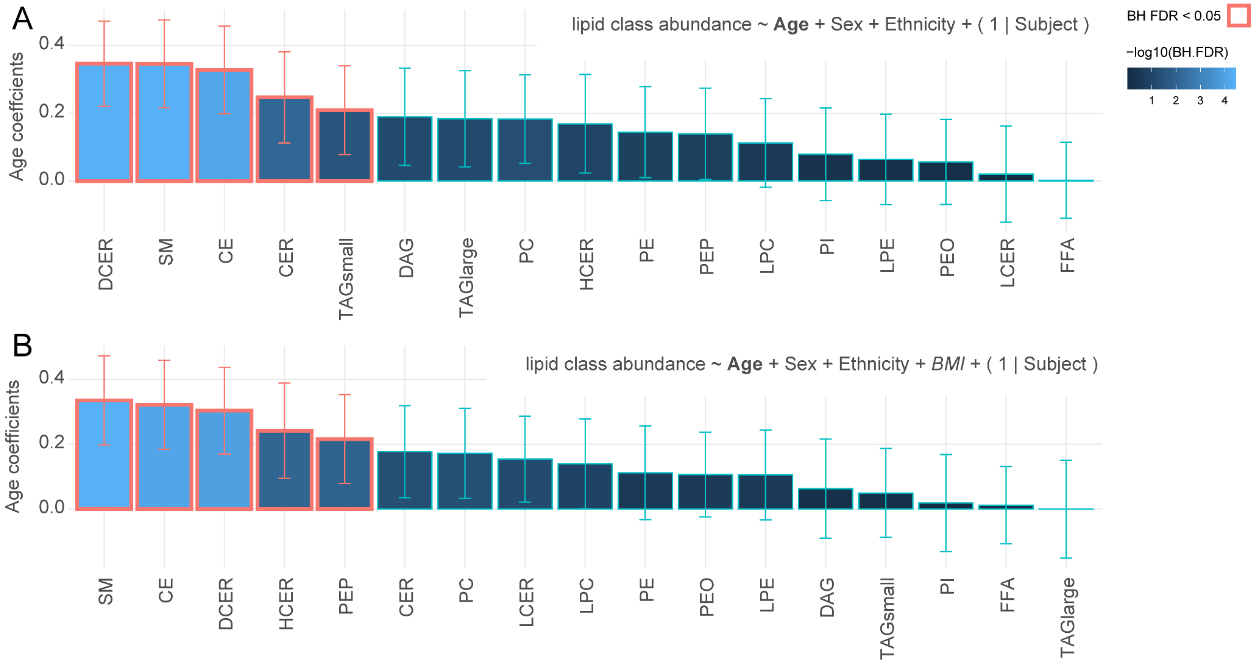

Supplementary Figure 11

Absolute aging models controlling (in contrast to delta age models used in Figure 5) for A) sex (fixed effect), ethnicity (fixed effect), and subject (random effect) as well as, B) in addition BMI (fixed effect). Age effect is estimated based on 736 lipid species aggregated (median) to their respective subclass for ‘healthy’ timepoints. A linear mixed effects model is used (lme4, REML = F) on scaled and centered data (scale) and p-values are estimated (jtools) before applying a Benjamini & Hochberg FDR correction. Red border indicates FDR < 5%, error bars denote 95% confidence intervals. Some lipids class coefficients change (e.g., TAGs) when BMI is controlled in the model.

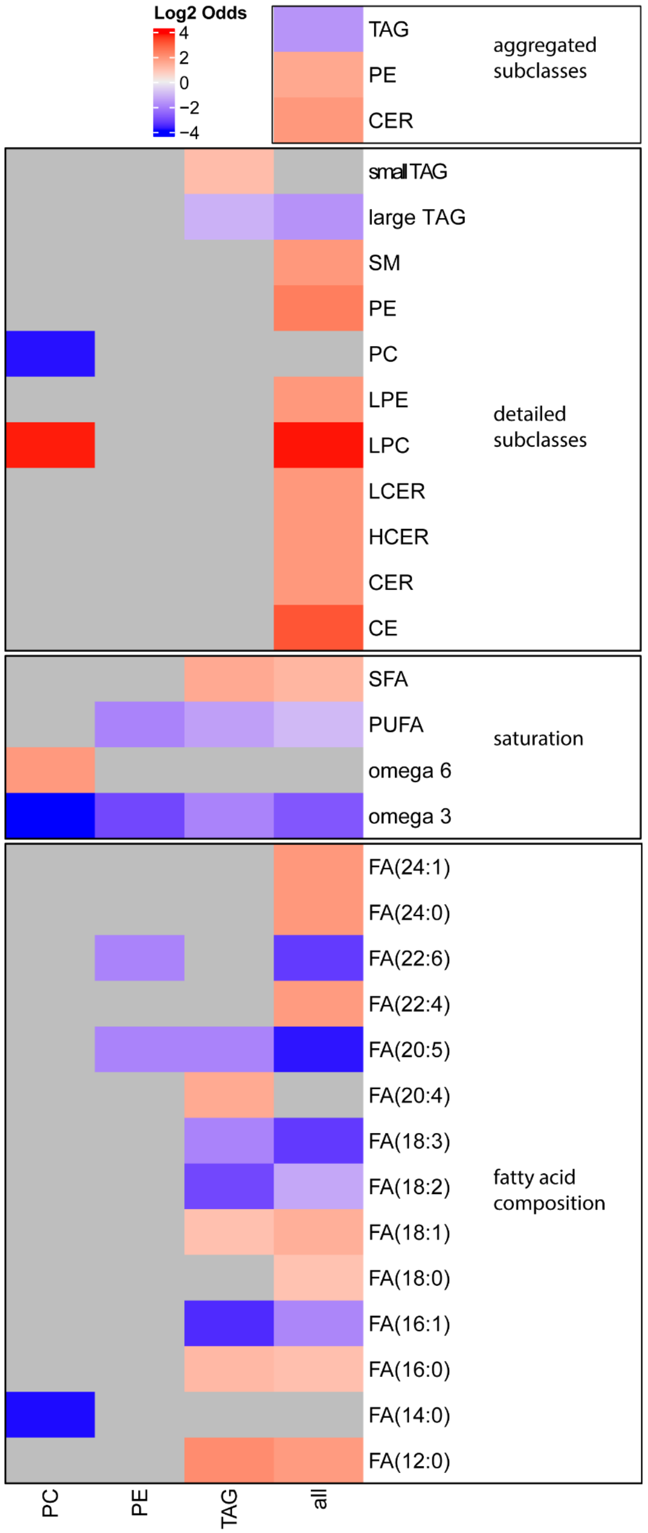

Supplementary Figure 12

Fisher's Exact test enrichment analysis comparing physicochemical properties associated with higher age (positive log2 Odds, red, determined for all positive delta age model coefficients at lipid species) to lower age (log2 Odds, blue, determined for all negative delta age model coefficients at lipid species). Annotations are based on physicochemical properties of individual lipid species. Enrichments are calculated for all lipids with negative and positive coefficients, respectively and independently within lipid subclasses, as well as across all ('all' column). Log2 Odds are depicted when respective annotation was significantly associated with lower or higher age (B.H. FDR < 5%). Infinite log2 Odds are imputed with the ½ * mean for positive / negative log2 Odds determined across all data.

Lipid - Cytokine and Chemokine Associations

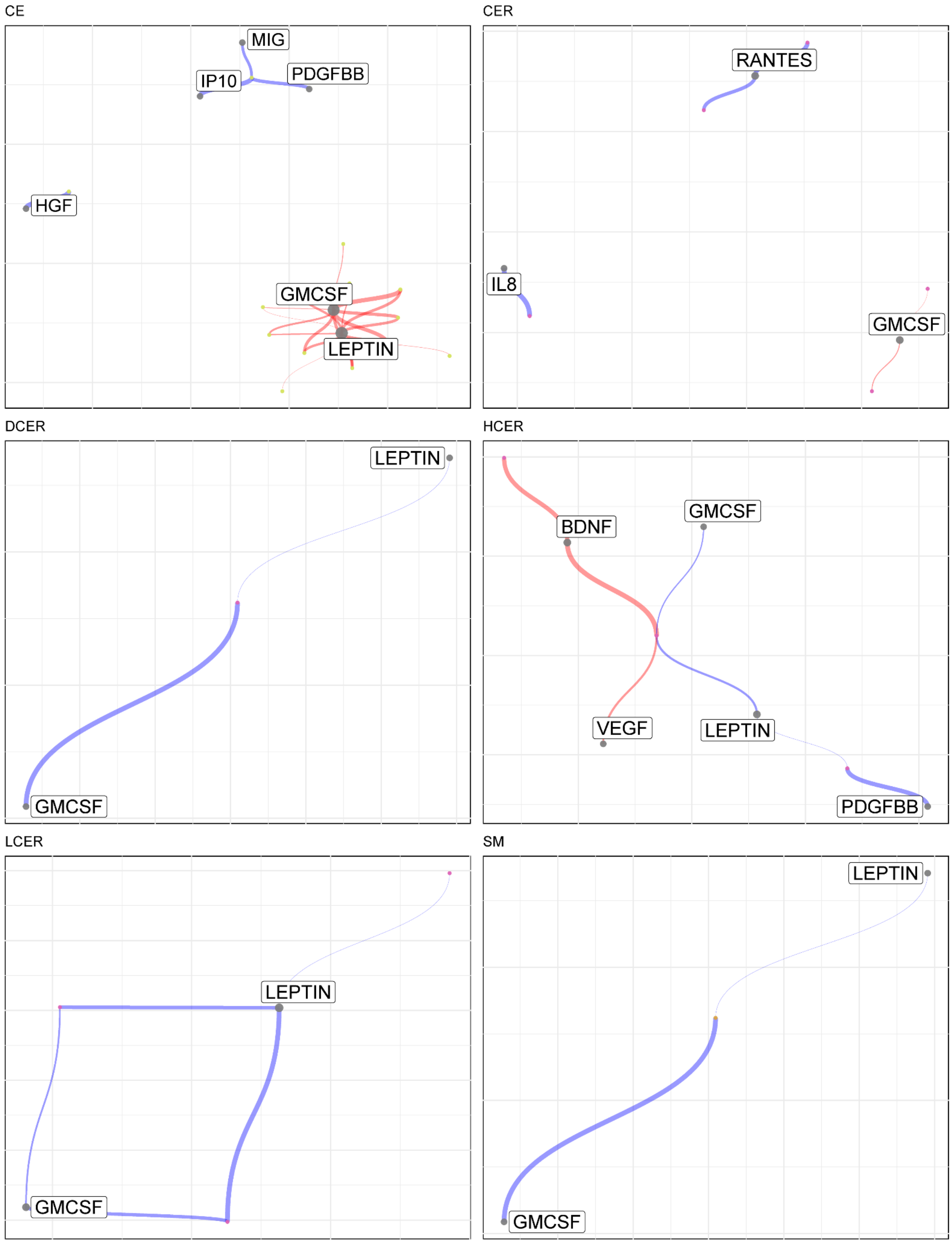

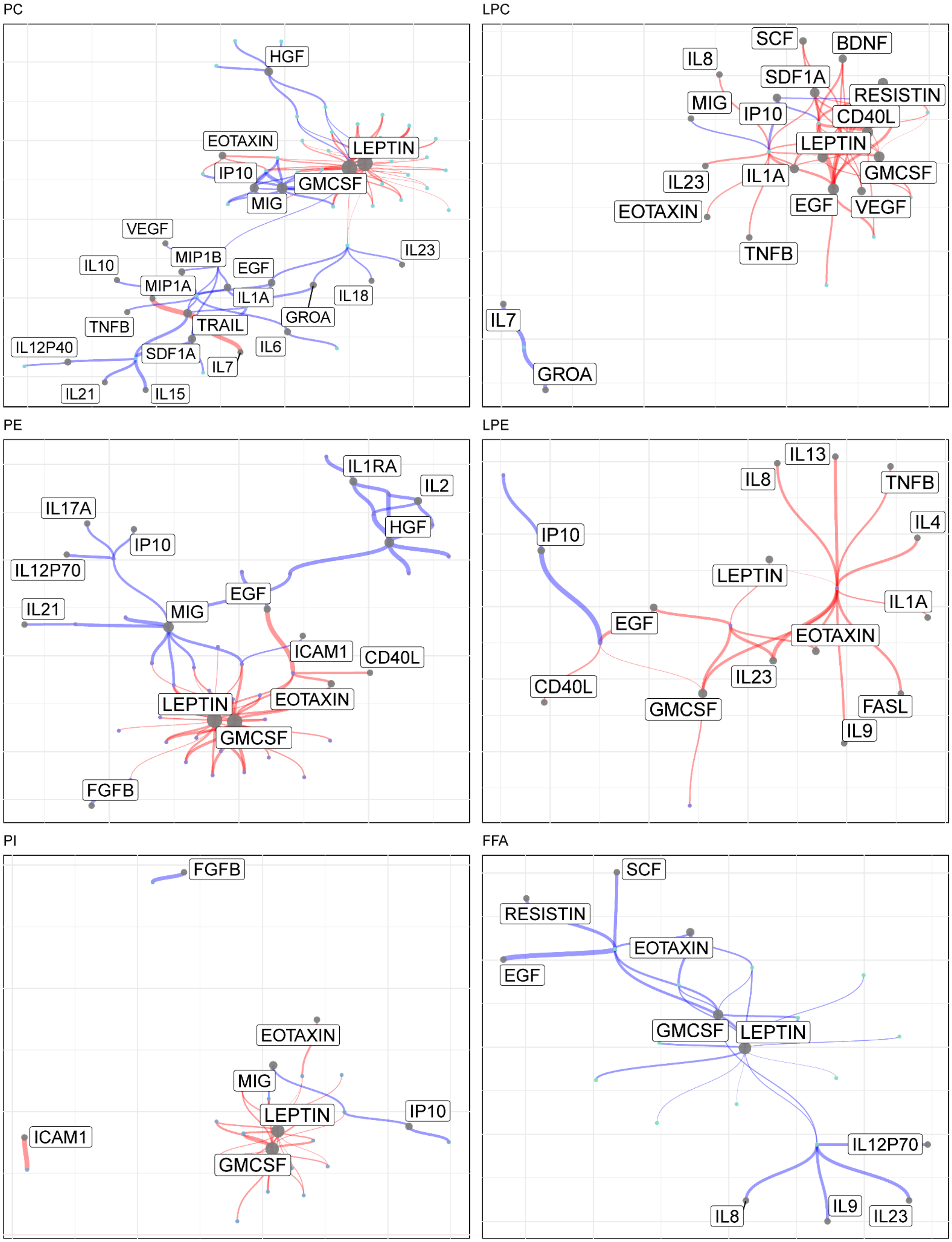
PC
HGF
EOTAXIN
LEPTIN
IP10
GMCSF
MIG
VEGF
MIP1B
IL23
IL10
EGF
MIP1A
IL1A
IL18
TNFB
TRAIL
GROA
IL12P40
SDF1A
IL6
IL7
IL21
IL15
LPC
SCF
BDNF
IL8
SDF1A
RESISTIN
MIG
IP10
CD40L
LEPTIN
IL23
IL1A
GMCSF
EOTAXIN
EGF
VEGF
TNFB
IL7
GROA
PE
IL1RA
IL2
IL17A
HGF
IP10
IL12P70
EGF
IL21
MIG
ICAM1
CD40L
LEPTIN
EOTAXIN
GMCSF
FGFB
LPE
IL13
IL8
TNFB
IL4
IP10
LEPTIN
IL1A
EGF
EOTAXIN
IL23
CD40L
GMCSF
FASL
IL9
PI
FGFB
EOTAXIN
MIG
LEPTIN
IP10
ICAM1
GMCSF
FFA
SCF
RESISTIN
EOTAXIN
EGF
GMCSF
LEPTIN
IL12P70
IL9
IL8
IL23

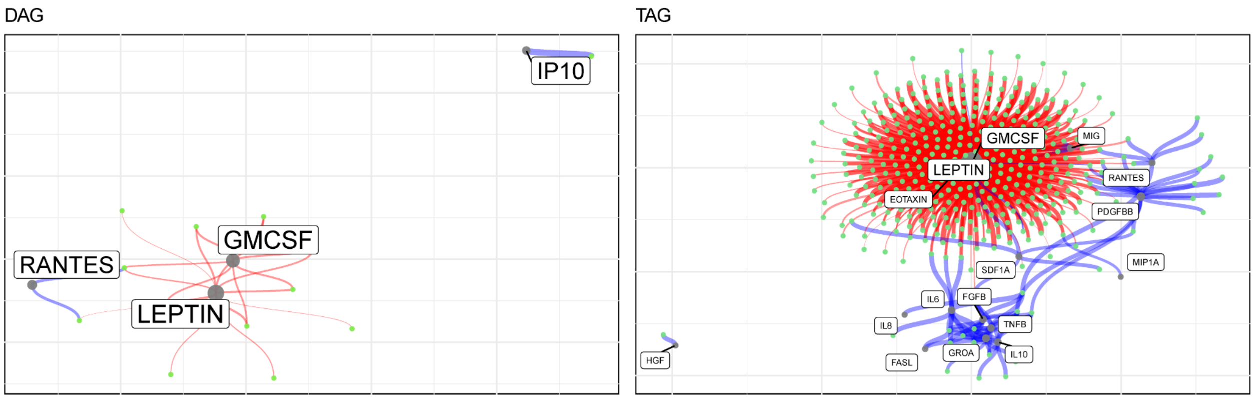

Supplementary Figure 13

Network of significant lipid species to cytokine associations indicating positive (red) and negative (blue) associations. Network was pruned based on the B.H. FDR of 5% for coefficients determined in linear mixed effects models (lme4, REML = F, on scaled and centered data (scale) and p-values are estimated (jtools)) for 736 lipids. Color indicates lipid class, edge width represents, absolute size of coefficient, and node size represents node connectivity (popularity). Network was assembled based on graph, layout = graphopt.

Mass Spectrometry Data Generation and Processing

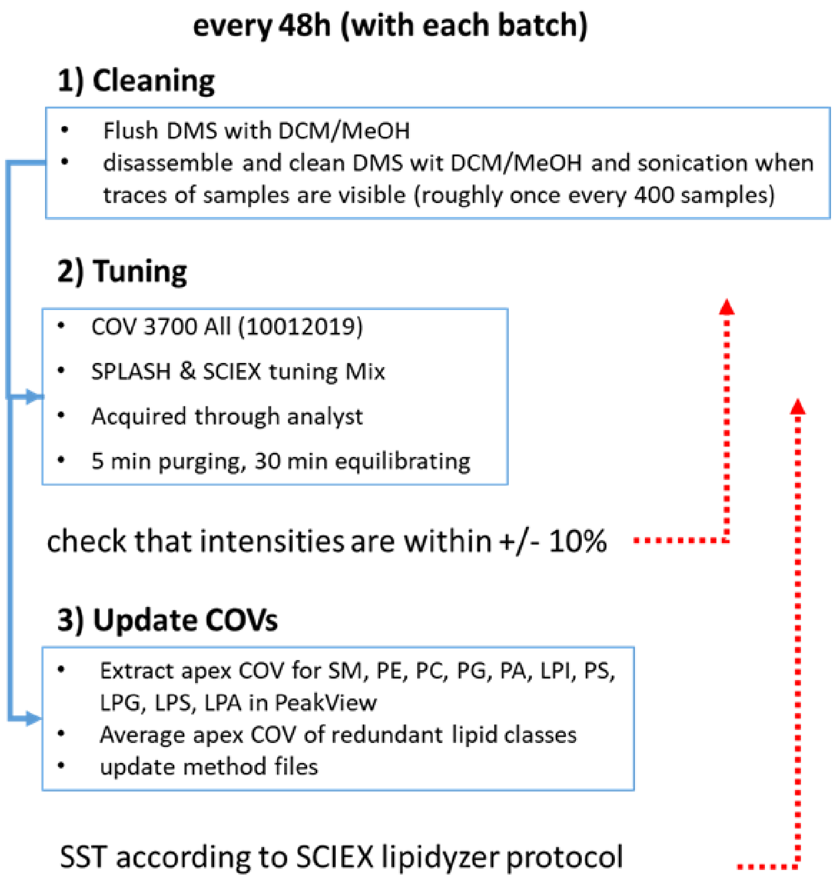

Supplementary Figure 14
Mass spectrometry acquisition and calibration workflow, detailing cleaning (1), tuning (2), and compensation voltage (COV) (3) calibration.

Supplementary Note 1: Lipid-Microbiome Associations

A growing body of evidence suggests that microbiome composition and activity play a central role in maintaining host metabolic-homeostasis. Altered composition of microbial populations (dysbiosis) is associated with conditions like inflammatory bowel disease and T2D, which affect host physiology at multiple levels including the immune system, energy metabolism, and gut permeability [1,2]. The association of distinct microbes with metabolites has been investigated extensively [3–5], but associations with different classes of lipids has not been investigated in detail.

We found that genera belonging to the class of *Clostridia* are mostly negatively correlated with lipids species. The preventive role of class *Clostridia* in absorption of intestinal lipids was previously reported in mice [6], and our data implies this relationship may also be true in humans. We previously reported a significantly lower level of total TAG among subjects with lower class *Clostridia* [7]. Here, the detailed quantitative profilings of lipid species enabled us to pinpoint the interactions to several specific TAG species and the bacterial family *Oscillospiraceae*. Furthermore, family *Clostridiaceae* showed negative correlation with (L)PE, PC, and CE, including CE(20:4) containing arachidonic acid, a fatty acid known to promote and modulate inflammation [8,9]. This indicates that specific microbes may modulate inflammation through preventing proinflammatory-lipid absorption.

Supplementary Note 2: Lipid Outlier Analysis

To further examine the utility of lipids to evaluate individuals' health states we performed an outlier analysis in which we identified participants that showed significant differences in their average lipid species and lipid class profiles compared to the population average (**Supplementary Figure 6**). For all participants, outliers within a particular lipid class were consistently either elevated or reduced, except for participant ZI2HHJI that has mixed outliers within DAGs. Some of the individuals in this study were diagnosed with chronic diseases or were taking medications known to impact lipids, such as statins. Although not all outlier lipids can be mapped to specific underlying chronic morbidities, we identified some known and potentially new disease associations which are of potential clinical applicability. For example, participant ZTJ7L7Z was diagnosed with hypertension, elevated cholesterol (>300 mg/dL), elevated blood triglycerides, elevated LDL, and was intolerant of statins. We also detected elevated outliers for CE, TAG, and DAG as well as elevated lipids in the CER and SM families, two lipid classes that accumulate in hepatitis steatosis [10]. This subject was later diagnosed with a mild hepatic steatosis (fatty liver) at a time point after sample collection had concluded, indicating that not only could lipid profiling reveal this condition at an early time point, but that many classes of lipids were dysregulated in this patient.

The participant ZTXY83G had the highest number of TAG and DAG outliers, consistent with a clinically diagnosed hyperlipidemia. Surprisingly, for the same subject, one circulating SM (SM 26:1) was significantly reduced, a pattern which has been reported to be associated with a lower risk of atherosclerosis [11,12]. The decrease in circulating SMs could be due to the participant's cholesterol-lowering statins (Lipitor) medication, which may lower SM [13]; however the elevated TAGs and DAGs in this individual was unexpected, suggesting potential subject-specific statin activity in lipid regulation. Participants ZWLGEWL was diagnosed with asthma, glaucoma, hypertension, and prediabetes and displayed reduced LPE and LPC levels. The negative correlation of LPC with CRP (**Figure 2D**) is consistent with elevated inflammation and CRP levels (average of 9.9 mg/L across 6 visits) in this individual. Finally, participant ZJOSZHK presented with an autoimmune vascular phenotype and was hospitalized for cerebral vascular spasm and multiple myocardial infarctions. Their lipid profile showed increased FFAs and lowered CEs, HCERs, and SMs suggesting novel lipid alterations associated with these pathophysiologies, although additional similar patients would need to be profiled to determine whether the lipid patterns observed stem from the autoimmune vascular phenotype. Overall, these outlier results suggest links between distinct lipid alterations with different clinical pathologies which ultimately may be valuable for subtyping these conditions and utility in early disease detection.

Supplementary References

1. Forbes, J. D., Van Domselaar, G. & Bernstein, C. N. The Gut Microbiota in Immune-Mediated Inflammatory Diseases. *Frontiers in Microbiology* vol. 7 Preprint at https://doi.org/10.3389/fmicb.2016.01081 (2016).
2. Gurung, M. *et al.* Role of gut microbiota in type 2 diabetes pathophysiology. *EBioMedicine* **51**, 102590 (2020).
3. Levy, M., Thaiss, C. A. & Elinav, E. Metabolites: messengers between the microbiota and the immune system. *Genes Dev.* **30**, 1589–1597 (2016).
4. He, Y. *et al.* Gut microbial metabolites facilitate anticancer therapy efficacy by modulating cytotoxic CD8 T cell immunity. *Cell Metab.* **33**, 988–1000.e7 (2021).
5. Agus, A., Clément, K. & Sokol, H. Gut microbiota-derived metabolites as central regulators in metabolic disorders. *Gut* **70**, 1174–1182 (2021).
6. Petersen, C. *et al.* T cell-mediated regulation of the microbiota protects against obesity. *Science* **365**, (2019).
7. Zhou, X. *et al.* Longitudinal Analysis of Serum Cytokine Levels and Gut Microbial Abundance Links IL-17/IL-22 With and Insulin Sensitivity in Humans. *Diabetes* **69**, 1833–1842 (2020).
8. Tallima, H. & El Ridi, R. Arachidonic acid: Physiological roles and potential health benefits - A review. *J. Advert. Res.* **11**, 33–41 (2018).
9. Dennis, E. A. & Norris, P. C. Eicosanoid storm in infection and inflammation. *Nature Reviews Immunology* vol. 15 511–523 Preprint at https://doi.org/10.1038/nri3859 (2015).
10. Hajduch, E., Lachkar, F., Ferré, P. & Foufelle, F. Roles of Ceramides in Non-Alcoholic Fatty Liver Disease. *J. Clin. Med. Res.* **10**, (2021).

11. Iqbal, J., Walsh, M. T., Hammad, S. M. & Hussain, M. M. Sphingolipids and Lipoproteins in Health and Metabolic Disorders. *Trends Endocrinol. Metab.* **28**, 506–518 (2017).
12. Chung, R. W. S. *et al.* Effect of long-term dietary sphingomyelin supplementation on atherosclerosis in mice. *PLoS One* **12**, e0189523 (2017).
13. Zhou, Q., Luo, A. & Kummerow, F. A. Lovastatin reversed the enhanced sphingomyelin caused by 27-hydroxycholesterol in cultured vascular endothelial cells. *Biochem Biophys Rep* **5**, 127–133 (2016).
