# Supplementary material for: Dynamic lipidome alterations associated with human health, disease and ageing
Source: Nat Metab. 2023 Sep 11;5(9):1578–94. doi: 10.1038/s42255-023-00880-1 (PMC10513930; doi:10.1038/s42255-023-00880-1)
Supplement: Supplementary file 2 — Reporting Summary [file 42255_2023_880_MOESM2_ESM.pdf]

nature portfolio

Corresponding author(s): Michael P. Snyder

Last updated by author(s): Jun 22, 2023

Reporting Summary

Nature Portfolio wishes to improve the reproducibility of the work that we publish. This form provides structure for consistency and transparency in reporting. For further information on Nature Portfolio policies, see our Editorial Policies and the Editorial Policy Checklist.

Statistics

For all statistical analyses, confirm that the following items are present in the figure legend, table legend, main text, or Methods section.

n/a	Confirmed	
☐	☒	The exact sample size (n) for each experimental group/condition, given as a discrete number and unit of measurement
☐	☒	A statement on whether measurements were taken from distinct samples or whether the same sample was measured repeatedly
☐	☒	The statistical test(s) used AND whether they are one- or two-sided<br>*Only common tests should be described solely by name; describe more complex techniques in the Methods section.*
☐	☒	A description of all covariates tested
☒	☐	A description of any assumptions or corrections, such as tests of normality and adjustment for multiple comparisons
☐	☒	A full description of the statistical parameters including central tendency (e.g. means) or other basic estimates (e.g. regression coefficient) AND variation (e.g. standard deviation) or associated estimates of uncertainty (e.g. confidence intervals)
☐	☒	For null hypothesis testing, the test statistic (e.g. F, t, r) with confidence intervals, effect sizes, degrees of freedom and P value noted<br>*Give P values as exact values whenever suitable.*
☒	☐	For Bayesian analysis, information on the choice of priors and Markov chain Monte Carlo settings
☒	☐	For hierarchical and complex designs, identification of the appropriate level for tests and full reporting of outcomes
☐	☒	Estimates of effect sizes (e.g. Cohen's d, Pearson's r), indicating how they were calculated

Our web collection on statistics for biologists contains articles on many of the points above.

Software and code

Policy information about availability of computer code

Data collection	MS data was collected using Analyst software which is an integral part of SCIEX QTrap and Triple Quad systems. In addition Lipidyzer Workflow Manager was used for MS data Acquisition. .wiff files were converted to *.mzML with MSConvert v3.0.
Data analysis	For downstream data processing R version 4.2.2 as well as Python 3.7 were employed including the packages dbnorm(v0.2.2), sva (v3.38.0), mzR (version 2.6.2), 'Rtsne' (v0.15), WGCNA (v1.70-3), stats (version 3.6.2), OmicsLonDA (v1.15.0), AnnoCrawler, lme4 (v1.1-27.1), ggraph (v2.1.0), igraph (v1.5.0), and tidygraph (v1.2).

For manuscripts utilizing custom algorithms or software that are central to the research but not yet described in published literature, software must be made available to editors and reviewers. We strongly encourage code deposition in a community repository (e.g. GitHub). See the Nature Portfolio guidelines for submitting code & software for further information.

Data

Policy information about availability of data

All manuscripts must include a data availability statement. This statement should provide the following information, where applicable:

- Accession codes, unique identifiers, or web links for publicly available datasets
- A description of any restrictions on data availability
- For clinical datasets or third party data, please ensure that the statement adheres to our policy

Processed lipid data are provided as additional Supplementary Data 1. Raw mass spec data are hosted on our portal at http://hmp2-data.stanford.edu/index.php under the substudy iPOP lipidomics as well as at https://www.metabolomicsworkbench.org/ under the direct link https://doi.org/10.21228/M8ZM5P. Cytokine as well as microbiome data are hosted at http://hmp2-data.stanford.edu/index.php. Lipids were classified partially based on their physicochemical properties reported in LION database (65).

Field-specific reporting

Please select the one below that is the best fit for your research. If you are not sure, read the appropriate sections before making your selection.

☒ Life sciences ☐ Behavioural & social sciences ☐ Ecological, evolutionary & environmental sciences

For a reference copy of the document with all sections, see nature.com/documents/nr-reporting-summary-flat.pdf

Life sciences study design

All studies must disclose on these points even when the disclosure is negative.

Sample size	Sample size was limited by number of subject in the study and number of samples collected from each subject.
Data exclusions	To ensure the accuracy and reliability of our analysis, we implemented several data filtering criteria. Firstly, we excluded biosamples with more than 25% missing data from analysis. Additionally, lipids with less than 10% valid values, as determined by the Lipidyzer reporting requirements, were also excluded. To further ensure high-quality quantitative results for the results presented in Figures 2-6, we removed any lipid with a coefficient of variation (CV) greater than 20% in QC samples and the few lipids for which the CV in QC samples was higher than across the remaining biosamples. Furthermore, due to limitations in separation by DMS, we did not include PAs in our analysis. We also excluded PS/LPS, PG/LPG from the analysis as they showed a significant number of missing values. PI(16:0,18:3) was removed from the dataset due to association with incorrect masses. Finally, QC_73 from batch 21 was removed due to separate clustering compared to all other QCs.
Replication	To evaluate reproducibility we included 104 quality control samples and show the corresponding reproducibility in the supplementary information. As mentioned above, a single QC sample was removed as it clustered independently.
Randomization	Participants were not randomized in this study. Samples were randomized for lipid extraction and mass spectrometry aquisition
Blinding	There was no blinding in this study because it is not relevant to the study (There is no allocation to groups or interventions in the study). Omics data was processed without knowledge of participants' clinical status.

Reporting for specific materials, systems and methods

We require information from authors about some types of materials, experimental systems and methods used in many studies. Here, indicate whether each material, system or method listed is relevant to your study. If you are not sure if a list item applies to your research, read the appropriate section before selecting a response.

Materials & experimental systems

n/a	Involved in the study	
☒	☐	Antibodies
☒	☐	Eukaryotic cell lines
☒	☐	Palaeontology and archaeology
☒	☐	Animals and other organisms
☐	☒	Human research participants
☒	☐	Clinical data
☒	☐	Dual use research of concern

Methods

n/a	Involved in the study	
☒	☐	ChIP-seq
☒	☐	Flow cytometry
☒	☐	MRI-based neuroimaging

Human research participants

Policy information about studies involving human research participants

Population characteristics	This cohort has been described in detail previously (Schüssler-Fiorenza Rose SM et al. 2019, Nature Medicine). In brief: The mean age of iPOP participants at time of enrollment was 53 years old and 51% were women. The self-reported ethnic distribution of the cohort was 60% European, 12% East Asian, 10% South Asian, 6% Jewish, 6% Hispanic, 6% African American, 6% Mixed/Other. The most common self-reported health conditions at entry were dyslipidemia (34%) and systemic hypertension (28%); participants were generally healthy. Over 78% of the cohort was overweight (BMI between 25 and 30) or obese (BMI ≥ 30). Our cohort which was enriched for DM risk, had a higher than normal family history of DM (55%). Family histories of systemic hypertension (54%), coronary artery disease (51%) and stroke (24%) were also common.
Recruitment	Participants were recruited via placement of advertisements in local newspapers and radio stations seeking “prediabetic volunteers” at risk for Type 2 diabetes for longitudinal multi-omic study. Screening in the CTRU entailed history and physical, anthropometric measurements, and fasting blood tests for exclusions including presence of anemia defined as hematocrit < 30, renal disease defined as creatinine > 1.5, history of any cardiovascular, malignancy, chronic inflammatory, psychiatric disease, and history of any bariatric surgery or liposuction. Sex was considered in the study design to increase balance of the demographics. Sex of participants refers to their biological (chromosomal) attributes. Gender is not specified. The overall

selection puts emphasis on western dietary and lifestyle associated morbidities. Moreover, the unique characteristics of the Stanford University surrounding community and that there may have been a self selection bias of people interested in intensive monitoring of health may have impacted their response to such monitoring increasing motivation to lifestyle changes.

Ethics oversight	Stanford University Institutional Review Board (IRB 23602)

Note that full information on the approval of the study protocol must also be provided in the manuscript.
